# Proliferation and migration of ML1 follicular thyroid cancer cells are inhibited by IU1 targeting USP14: role of proteasome and autophagy flux

**DOI:** 10.3389/fcell.2023.1234204

**Published:** 2023-08-30

**Authors:** Vignesh Srinivasan, Muhammad Yasir Asghar, Sadia Zafar, Kid Törnquist, Dan Lindholm

**Affiliations:** ^1^ Medicum, Department of Biochemistry and Developmental Biology, Medical Faculty, University of Helsinki, Helsinki, Finland; ^2^ Minerva Foundation Institute for Medical Research, Helsinki, Finland; ^3^ Cell and Tissue Dynamics Research Program, Institute of Biotechnology, Helsinki Institute of Life Sciences, University of Helsinki, Helsinki, Finland; ^4^ Applied Tumor Genomics Research Program, Research Programs Unit, University of Helsinki, Helsinki, Finland; ^5^ Department of Pathology, HUSLAB, HUS Diagnostic Center, University of Helsinki, Helsinki University Hospital, Helsinki, Finland; ^6^ Faculty of Science and Engineering, Cell Biology, Åbo Akademi University, Turku, Finland

**Keywords:** USP14, proteasome, autophagy, thyroid cancer, cell proliferation, cell migration

## Abstract

USP14 is a deubiquitinating enzyme involved in protein degradation by interacting with the proteasome and removal of poly-ubiquitin chains on target proteins. USP14 can influence cellular processes such as cell survival, DNA repair, ER stress, endocytosis, and the inflammatory response. USP14 further plays a role in tumor growth, and the inhibition of USP14 by compounds such as IU1 may affect cancer cell migration and invasion. Here we have studied the mechanisms for the action of IU1 in ML1 follicular thyroid cancer cells, comparing them with control, primary thyroid cells. Treatment with IU1 reduced proliferation of ML1 cells in a concentration-dependent manner, and more prominently than in control cells. IU1 decreased basal migration of ML1 cells, and after stimulation of cells with the bioactive compound, sphingosine-1-phosphate. The sphingosine-1-phosphate receptor 3 was increased in ML1 cells as compared with control thyroid cells, but this was not influenced by IU1. Further studies on the mechanism, revealed that IU1 enhanced the proteasome activity as well as LC3B-dependent autophagy flux in ML1 cells with an opposite effect on control thyroid cells. This indicates that IU1 elicits a cell-type dependent autophagy response, increasing it in ML1 cancer cells. The IU1-mediated stimulation of autophagy and proteasomes can likely contribute to the reduced cell proliferation and migration observed in ML1 cells. The precise set of proteins affected by IU1 in ML1 thyroid and other cancer cells warrant further investigations.

## Introduction

Protein degradation is a tightly regulated process to maintain cell and tissue homeostasis that is important for cell viability. The ubiquitin-proteasome system and the lysosomal-autophagy machinery are the two main pathways for protein degradation. USP14 is a deubiquitinating enzyme associated with the proteasome that is a critical regulator of protein degradation by removing lysine 48-linked polyubiquitin chains on protein substrates (Crimmins et al., 2006; Lee et al., 2016; Kim and Goldberg, 2017). Recent findings further show that USP14 can influence other cellular processes, including autophagy and ER-stress (Hyrskyluoto et al., 2014; Srinivasan et al., 2020).

In view of its importance for cell viability and proliferation, strategies targeting USP14 have been explored with the aim to influence protein degradation in cancer cells. Work from different laboratories have focused on 1-[1-2,5-dimethyl-1H-pyrrol-3-yl]-2-pyrrolidin-1-ylethanone (IU1) that was identified as a potent and selective inhibitor of USP14 (Lee et al., 2010; Abramson, 2018). IU1 inhibits USP14 by binding to the catalytic cleft in USP14 thus blocking its enzyme activity (Lee et al., 2010). Recently, chemical derivatives of IU1 have been developed, such as IU1-47 and IU1-248, with a higher potency of inhibition (Boselli et al., 2017; Wang et al., 2018; Han et al., 2019). There are also other compounds, such as b-AP15 and its analog VLX157, used for inhibiting USP14, but they further target the ubiquitin C-terminal hydrolase-5 (UCHL5) that is also associated with the 19S proteasome (Paulus et al., 2016; Wang et al., 2016; Cai et al., 2017). Small-molecule inhibitors of USP14 may provide benefits in targeting tumor cells, but possible off-target effects are of major concerns with regard to treatments (Kiprowska et al., 2017).

In the present work, we have studied the effects of IU1 on cellular pathways for protein degradation using ML1 cancer cells as an important culture model for survival, and migration of thyroid tumors.

We have previously shown that the proliferation and migration of ML1 follicular thyroid cancer cells are affected by calcium signals by the bioactive lipid sphingosine 1-phosphate (S1P) and the expressional profile of S1P receptors (Balthasar et al., 2006; Kalhori et al., 2013; Kalhori et al., 2016; Asghar et al., 2018; Asghar et al., 2021).

We observed here that the expression of USP14 is downregulated in the ML1 thyroid cancer cells compared with primary thyroid cells. Addition of IU1 caused a significant decrease in cell proliferation and migration of ML1 cells in a dose dependent manner. IU1 also blocked the S1P induced cell migration of ML1 cells but did not significantly affect the levels of S1PR3 receptor. The mechanisms by which IU1 influence ML1 cells related to effects on the proteasome and autophagy processes in these cells.

## Methods

### Cell culture

Human primary thyroid cells were cultured in H6621 medium with supplements according to the manufacturer’s instructions. ML1 cells were cultured in DMEM with 10% FBS, 1% L-glutamine (L-Glu), and 1% penicillin/streptomycin (P/S). FTC-133 cells were cultured in DMEM:F-12 (Ham’s) medium (1:1) supplemented with 10% FBS and 1% L-Glu and 1% P/S. All cell cultures were maintained in a water-saturated atmosphere with a continuous supply of 5% CO_2_ and 95% air at 37 °C in the incubators. Cells were treated with different amounts of IU1 or with chloroquine diphosphate (CQ) under different conditions followed by immunoblotting or cell assays as described below.

### Cell viability and proliferation assays

To estimate the number of cells we used the 3-(4,5-dimethylthiazol-2-yl)-2,5-diphenyltetrazolium bromide (Sigma) MTT assay. Briefly, 10,000 cells were grown on 96 well plate for 24 h and then treated with DMSO (controls), mitomycin C, or 1–100 µM of IU1 for 24 h. MTT was added for 2 h and insoluble formazan substrate was then reconstituted in a solvent of 0.1 M HCl-isopropanol and incubated for 30 min with gentle agitation. Absorbance was measured at 560 nm and the value reflects the relative number of surviving cells following each treatment. The experiments were repeated more than three times.

To examine cell proliferation, we used radioactive [3H] thymidine added to the cells. For this, 50,000 cells were seeded on 35-mm plates and allowed to grow for 24 h. The cells were then treated with IIU1 as above for 24 h, and 0.4 μCi/mL [3H] thymidine was added to the cells for the last 4 h of incubation. The cells were washed three times with PBS, incubated for 10 min with 1 mL of 5% trichloroacetic acid, and then for 10 min with 1 mL of 0.1 M NaOH. The samples were transferred into scintillation vials and 3 mL of high sample load scintillation cocktail Optiphase Hisafe 3 was added in each sample. The radioactivity was measured using a Wallac 1414 liquid scintillation counter. The radioactivity data counts per minute (cpm) was normalized, and the results presented as % proliferation. The experiments were repeated more than three times.

### Cell migration assay

In these experiments, 50,000 cells were seeded onto collagen IV coated Transwell inserts in serum-free medium and allowed to migrate for 6 h towards 10% FBS containing medium in lower wells. To study the effect of bioactive lipid S1P, the cells were lipid starved by changing the medium to 0.2% FAF-BSA-containing serum-free medium (SFM) for overnight prior to the start of the migration assays. Next day, cells were allowed to migrate towards 10% lipid stripped FBS (chemoattractant) for 6 h in the presence or absence of S1P (100 nM). The non-invaded cells on the top of Transwell insert membranes were removed carefully with a cotton swab. The cells on the bottom side of the insert membrane were fixed with 2% paraformaldehyde for 10 min, followed by staining with 0.1% crystal violet (in 20% methanol) for 5 min. Next, the membranes were washed with PBS and water and allowed to dry for overnight. The stained cells were counted using ×40 magnification from eight microscopic fields per insert. The data were normalized and presented as % invasion.

### Immunoblotting

Cells were washed twice with ice-cold PBS and lysed in RIPA buffer (150 mM NaCl, 1% Triton-X-100, 0.5% sodium deoxycholate, 1% SDS, 50 mM Tris-HCl, pH 7.4) supplemented with protease inhibitor and phosphatase inhibitor (Phosphostop, Roche). Protein concentration was measured with the BCA protein assay kit (Pierce, Thermo fisher), and equal amounts of protein were loaded onto PAGE and blotted using nitrocellulose membrane filters (Sartorius). The membranes were incubated for 1 h in 5% skimmed milk or 5% bovine serum albumin, in TBS-T (50 mM Tris-HCl pH 7.5, 150 mM NaCl, 0.1% Tween 20) and then with the primary antibodies overnight at 4°C with gentle agitation. The primary antibodies used were as follows: anti-USP14 (1:2000, Sigma), anti-S1P1 receptor (1:1000), anti-S1P3 receptor (1:2000), Anti-K48 linked polyubiquitin (1:2000, CST), anti-LC3B (1:1000, CST), anti-GABARAP (1:1000, CST), anti-SQSTM1/p62 (1:3000, Sigma), anti–MDM2 (1:1000, CST), and anti-GAPDH (1:2000, Millipore), Membranes were further washed and incubated with HRP-conjugated secondary antibody (1:2500, Jackson Immunoresearch Laboratories) for 1 h at room temperature. Protein signals were detected using enhanced chemiluminescence substrate (Pierce Thermo Scientific). Immunoblots were quantified with ImageJ (NIH) quantification software (Hyrskyluoto et al., 2014; Srinivasan et al., 2020).

### Co-immunoprecipitation

Cells were lysed in a modified RIPA lysis buffer containing 50 mM Tris-HCl pH 7.7, 150 mM NaCl, 1% NP-40, and 0.5% sodium deoxycholate for 30 min on ice. Cell lysates were further centrifuged, supernatants were collected, and the protein concentration measured using BCA protein assay kit (Pierce, Thermo Fisher). Equal amount of protein lysate was taken and precleared, followed by incubation with Anti-USP14 (Sigma) overnight in a rotor at 4°C. The following day, BSA-blocked agarose beads were added to the lysates. Following 3-h incubation, beads were centrifuged and washed with lysis buffer. Antibody-protein conjugates were eluted by addition of 2X Laemmli buffer and heating 98°C, 5 min. The eluates were then subjected to immunoblotting for the indicated antibodies (Srinivasan et al., 2020).

### RNA isolation and quantitative PCR

RNA was extracted with Aurum™ Total RNA Mini Kit (Bio-Rad; CA, United States) according to the manufacturer’s instructions. RNA integrity was checked by gel electrophoresis and RNA concentration and purity was determined with Nanodrop 2000 (Thermo Fisher Scientific; Waltham, MA) and NanoVue Plus (Healthcare Bio-Sciences AB; Uppsala, SE). cDNA synthesis was performed with 1 µg of total RNA by using GoScript Reverse Transcriptase (Promega) according to manufacturer instructions, using Oligo (dT)15 primers (Promega) and 3 mM MgCl_2_. Reaction mixtures lacking either reverse transcriptase or RNA were used as negative controls. For USP14, the primers sequences were as follows: Forward, 5′-TGT​GCC​TGA​ACT​CAA​AGA​TGC-3′, and Reverse, 5′-ATA​TAC​TGC​GCT​GAA​GCC​ATT​T-3’. For GAPDH they were: Forward, 5′-GTT​CGA​CAG​TCA​GCC​GCA​TC-3′, and Reverse, 5′-GGA​ATT​TGC​CAT​GGG​TGG​A-3′ respectively. qPCR was performed under following conditions: 95°C for 2 min followed by 39 cycles of 95°C for 5 s and 60°C for 30 s. Subsequently, a melt curve analysis was performed from 65°C to 95°C, with 0.5°C increments and hold 5 s at each temperature. Amplification and detection of SYBR-Green were performed using BIO-RAD iQ SYBR Green Supermix in the CFX Duet Real-Time PCR system (Bio-Rad, Hercules, California, United States). Glyceraldehyde-3-phosphate dehydrogenase (GAPDH) was used as a reference and data was normalized using the ΔΔCt (threshold cycle) method. Values are presented as % gene expression and are mean of triplicate determinations. The experiments were repeated three independent times.

### Native-gel electrophoresis and in-gel proteasome activity assay

Nativel gel electrophoresis for in-gel 20S proteasome activity and immunoblotting was performed as described earlier with a few modifications (Elsasser et al., 2005; Roelofs et al., 2018; Yazgili et al., 2021). ML1 cells were lysed in OverKleeft (OK) lysis buffer (50 mM Tris-HCl pH7.5, 2 mM DTT, 5 mM MgCl_2_, 10% glycerol, 2 mM ATP, and 0.05% Digitonin) and incubated on ice for 20 min with vortexing in-between incubation. Following this, lysates were centrifuged at 27,670xg for 20 min, 4°C. Supernatants were collected and protein estimated with BCA assay. Equal amount of protein was taken, and lysates separated on a 4% poly-acrylamide native gel made utilizing a Tris-Borate buffer. Freshly made Tris-borate buffer containing EDTA-Na_2_, ATP and MgCl_2_ was used as the running buffer and the gels were separated at a constant voltage of 120 V for 2 h 30 min at 4°C. Following this, gel was carefully transferred into the buffer containing the substrate for in-gel activity assay.

The gel was incubated at 37°C for 15 min in a buffer containing 50 mM Tris-HCl pH7.4, 5mM MgCl_2,_ 1 mM ATP and 100 mM of Suc-Leu-Leu-Val-Tyr-AMC (Bachem) substrate for chymotrypsin-like activity of 20S CP. Following this, the gels were imaged at an excitation wavelength of 380 nm and emission wavelength of 460 nm. Upon imaging, the gels were incubated again for 15 min 37°C in the above-mentioned buffer with the addition of SDS to open the gating of free 20S CP. Following this the gels were imaged again to assess the activity of free 20S CP.

For immunoblotting of the native gels, upon separation the gels were transferred to Western blotting running buffer containing SDS to impart negative charge to complexes in the gel. The gels were incubated for 10 min in this buffer with gel shaking and then transferred to the blotting buffer containing methanol. Post-this, conventional immunoblotting transfer protocol was performed with transfer for 16 h, 20 V at 4°C followed by washes, blocking, and incubation with 20S (BML-PW8195, Enzo Lifesciences) or USP14 antibody (Sigma) antibodies.

### DUB assay using the Ub-VME substrate

The labelling assay for DUB activity was performed as described earlier with a few modifications (Borodovsky et al., 2001; Srinivasan et al., 2020). Following stimulation with IU1 the ML1 cells were lysed in 50 mM Tris (pH 7.4), 250 mM sucrose, 5 mM MgCl_2_, 1 mM DTT and 1 mM ATP for 1 h at 4°C. 50 μg of lysates was then incubated with 3 mM ubiquitin vinyl methyl ester (HA tag; Enzo Life Science) for 3 h at 37°C. The samples were then boiled 95°C for 5 min in denaturing Laemmli buffer and analyzed by immunoblotting with USP14 antibody.

### Statistical analyses

Statistical comparison was performed using Student’s t-test or one-way/two-way ANOVA followed by a Bonferroni *post hoc* test, depending on the experimental design. *p*-value, *p* < 0.05 was considered as statistically significant. The statistical analysis and graph design was performed using GraphPad PRISM software.

## Results

### USP14 levels are reduced in ML1 thyroid cancer cells

**FIGURE 1:**
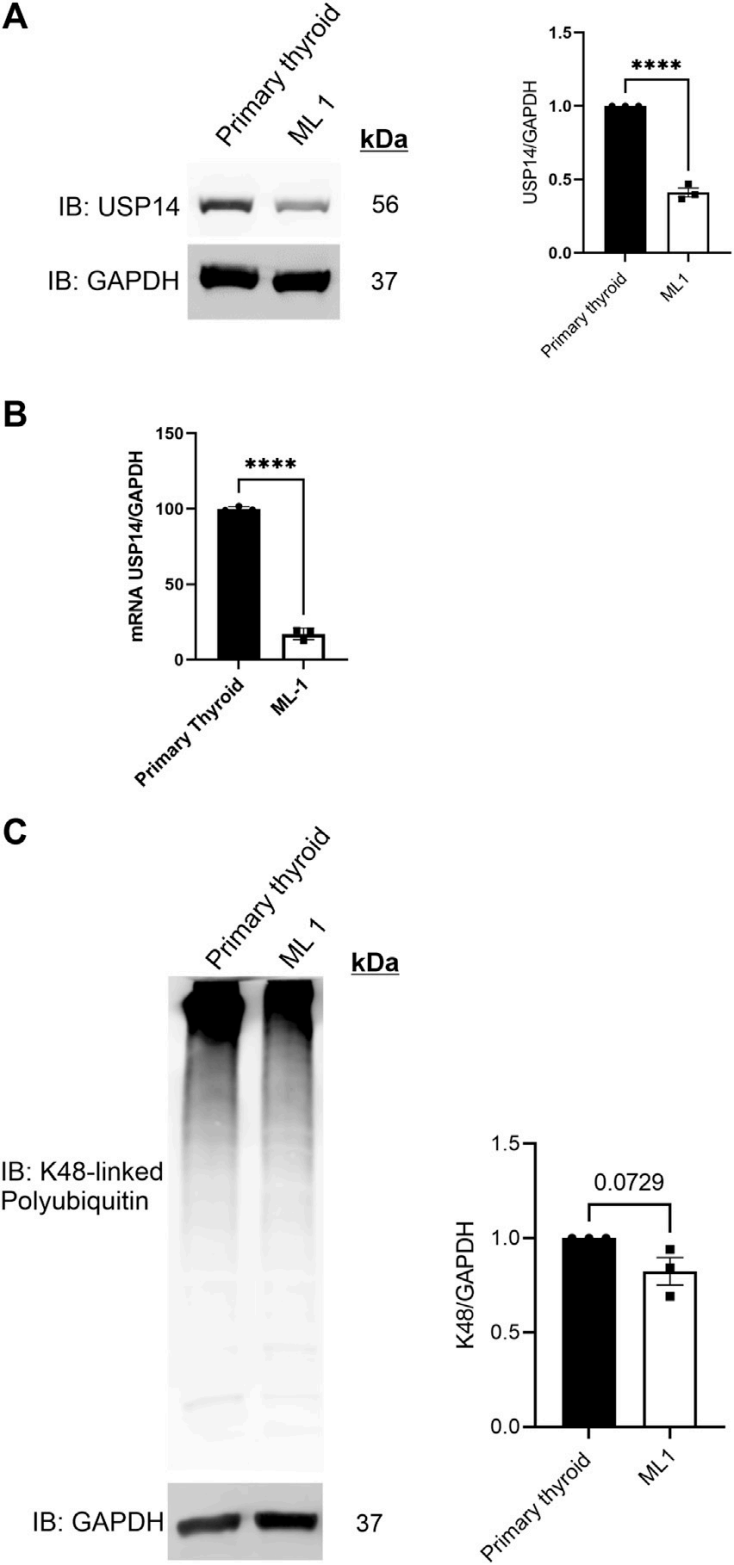
USP14 levels are reduced in ML1 thyroid cancer cells. Primary thyroid cells and ML1 cells were cultured *in vitro* and analyzed as below. **(A)** Immunoblotting for USP14 was done as described in Methods. Right panel represents histogram of the densitometry ratio of USP14 normalized to GAPDH. **(B)** qPCR was done as described in Methods. Histogram represents the expression levels of USP14 normalized to GAPDH. Control set to 100. *n* = 3, *p*-value was calculated by Student’s t-test. *- *p* ≤ 0.05, **- *p* ≤ 0.01, ***- *p* ≤ 0.001, ****- *p* ≤ 0.0001. **(C)** K48-linked polyubiquitin was estimated as described in Methods. Right panel represents the histogram of the densitometry ratio of K48-linked PolyUb to GAPDH.

Alterations in cellular levels of USP14 levels have been associated with different types of cancer (Didier et al., 2018; Han et al., 2019; Liu et al., 2019; Geng et al., 2020). To investigate the expression of USP14 in thyroid cancer cell lines, we employed immunoblotting revealing that the protein levels of USP14 was lower in ML1 thyroid cancer cells compared with primary thyroid cells (Figure 1A). Furthermore, using quantitative PCR we observed a reduced expression of USP14 in ML1 cells compared with controls (Figure 1B). Together this shows that USP14 is decreased in the thyroid cancer cells, likely due to a reduced gene expression.

Reduced levels of USP14 in ML1 cells suggests possible changes in proteasome functions. To study this, we analyzed the levels of K48-linked polyubiquitin chains (polyUb) in the cells, however, there was no differences between ML1 cells compared with primary cells (Figure 1C).

### ML1 cells exhibit increased autophagic flux

**FIGURE 2:**
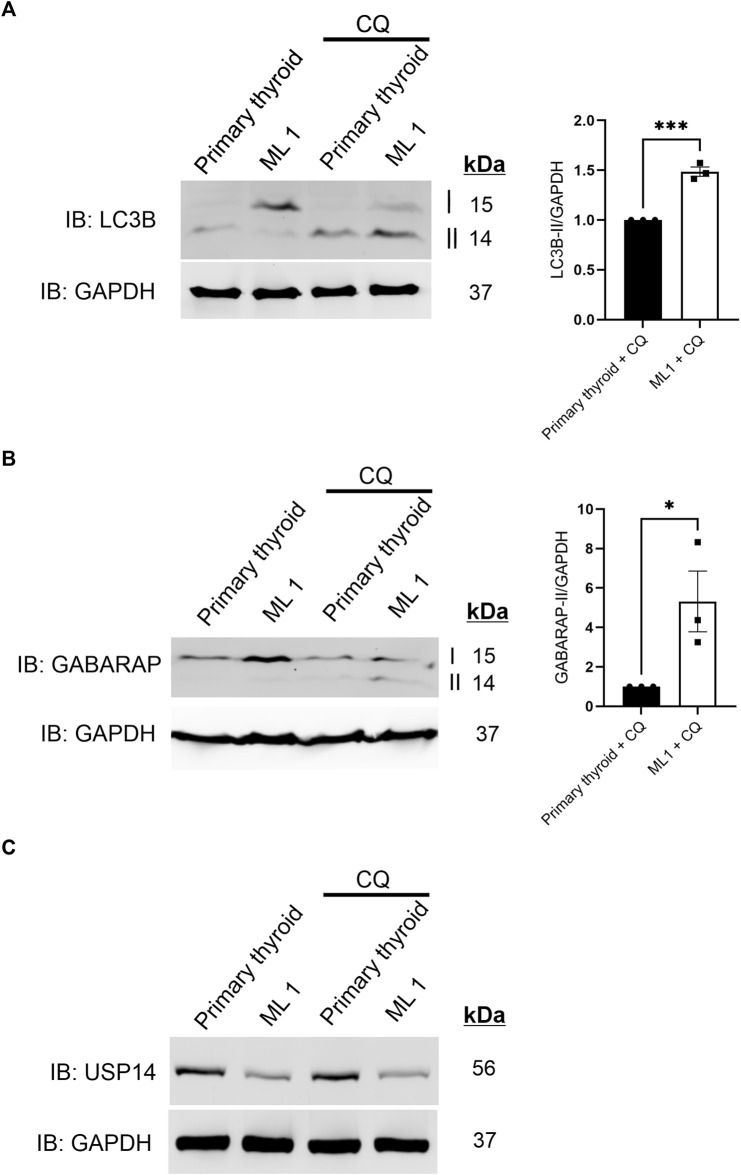
ML1 cells exhibit increases in LC3B and GABARAP-dependent autophagy flux. Primary thyroid and ML1 cells were stimulated with chloroquine diphosphate 100 µM CQ for 4 h to inhibit the autophagy flux and subjected to immunoblotting as above. **(A)** LC3B, right panel represents histogram of the densitometry ratio of LC3B-II normalized to GAPDH. **(B)** GABARAP, right panel represents histogram of the densitometry ratio of GABARAP-II normalized to GAPDH. *n* = 3, *p*-value was calculated by Student’s t-test. *- *p* ≤ 0.05, **- *p* ≤ 0.01, ***- *p* ≤ 0.001. **(C)** USP14.

Given that there was no clear difference in K48-linked polyUb levels, we next investigated whether autophagy could be changed in the ML1 cells compared with primary thyroid cells. During autophagy, the Microtubule-associated protein 1A/1B-light chain 3 (LC3B) and Gamma-aminobutyric acid receptor-associated protein (GABARAP), are processed and converted to LC3B-II and GABARAP-II and these can be revealed using immunoblots. We observed that the conversion of LC3B is relatively lower in ML1 cells compared with primary thyroid cells (Figure 2A) indicating a lower rate of LC3B lipidation and less autophagy. For GABARAP the situation was similar though it was hard always to detect the GABARAP-II in the immunoblots (Figure 2B). To study the process of autophagy in more detail, we employed chloroquine (CQ) that inhibits the fusion of autophagy vesicles with lysosomes and can be taken as an index for estimating the autophagy flux (Klionsky et al., 2016). Addition of CQ increased the levels of LC3B-II (Figure 2A) and of GABARAP-II (Figure 2B) in the cells and more so in ML1 cells than in primary thyroid cells. Together these results demonstrate that the ML1 cells exhibit changes in autophagy compared with primary thyroid cells with an increase in autophagy flux.

Previous reports have suggested that USP14 itself could be a substrate for autophagy (Sharma et al., 2020). However, we noted no changes in USP14 levels in the thyroid cells upon inhibition of autophagic flux by CQ (Figure 2C), indicating that USP14 itself is not regulated by autophagy either in the ML1 or in the primary thyroid cells.

### IU1 inhibits proliferation and migration of ML1 cells

**FIGURE 3:**
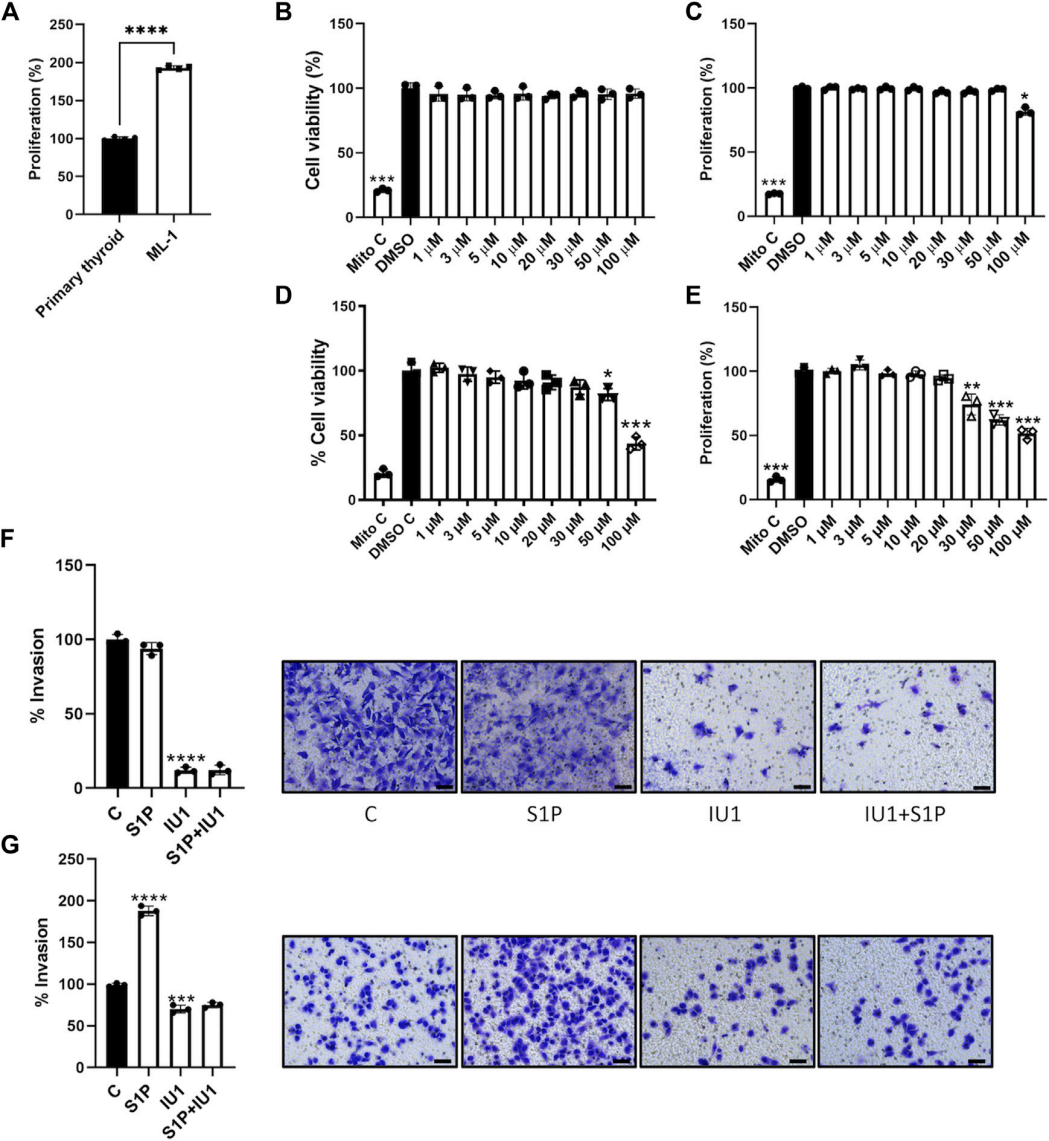
IU1 inhibits the proliferation and S1P-evoked migration of ML1 thyroid cancer cells **(A)** ML1 cancer cells and primary thyroid cells were cultured for 48 h in the presence of radioactive thymidine added for the last 4 h as described in Methods. Cell proliferation was higher in ML1 cells compared with control thyroid cells. Values are means ± S.E.M, *n* = 3. ****p < 0.0001. **(B,C)** Primary thyroid cells were cultured in the absence or presence of 1–100 µM IU1 for 24 h. Cell viability was estimated by the MTT assay as described in Methods **(B)**, and cell proliferation was determined by thymidine labelling as above **(C)**. Mitomycin was employed as an internal control for cell proliferation. Values are means ± S.E.M. and normalized to respective controls without IU1. *n* = 3. *p < 0.05 **(D,E)** ML1 thyroid cancer cells were cultured in the absence or presence of 1–100 µM IU1 for 24 h Cell viability was estimated by the MTT assay **(D)**, and cell proliferation was determined by thymidine labelling **(E)**. Values are mean ± S.E.M and normalized to respective controls without IU1 *n* = 3. *p < 0.05, **p < 0.01, ***p < 0.001. **(F,G)** Cell migration was investigated using Transwell chambers as described in Methods in the absence or presence of 20 µM IU1. Primary thyroid cancers cells are shown in **(F)** and ML1 thyroid cancer cells in **(G)**. IU1 decreased basal migration observed in control and ML1 cells. And abolished the migration induced by 100 nM S1P in ML1 cells. Values are means ± S.E.M, *n* = 3. ***p < 0.001, ****p < 0.0001. Representative images of cells in Transwell inserts and stained with crystal violet are shown to the right. The scale bar is 50 µm.

We reasoned that the reduced levels of USP14 in ML1 cells could afford an advantage for using USP14 inhibitors, such as IU1 (Liu et al., 2019) as the tumor cells may be more sensitive to its action compared with control cells. First, we measured the proliferation rate of the primary thyroid cells and the ML1 cells. We treated ML1 and primary thyroid cells with different concentrations of IU1 and examined them further. The proliferation rate was higher in ML1 cells compared with controls (Figure 3A). Data further showed that IU1 decreased cell number, as observed in the MTT assay for cell viability (Figures 3B, C), and the amount of DNA synthesis, as measured by H^3^-thymidine incorporation, in the ML1 cell cultures (Figures 3D, E). The effects of IU1 on cell proliferation were concentration dependent and became evident with concentrations higher than 20 µM of the compound (Figures 3D, E). Compared with ML1 cells the primary thyroid cells were less sensitive to IU1. Thus, the number of cells remained largely constant after treatment with IU1 (Figure 3B), whereas the DNA synthesis was reduced only at the highest concentration of 100 µM (Figure 3C). Next, we examined whether IU1 may influence cell migration/invasion by treating the cells in Transwell migration chambers. For this we choose, 20 µM IU1 that was neither toxic to primary thyroid cells, nor overtly so to the ML1 cells (Figures 3B–E). The results show that 20 µM IU1 significantly reduced the number of both primary thyroid cells and ML1 cells migrating in the Transwell chamber within 24 h (Figures 3F, G). This demonstrates that IU1 has the capacity to reduce cell migration of both ML1 and primary thyroid cells rather efficiently.

To investigate the underlying mechanisms, we turned to S1P that is a bioactive phospholipid with large effects on cell proliferation and migration (Pyne and Pyne, 2010; Monteith et al., 2017; Asghar et al., 2018; Janneh and Ogretmen, 2022). Addition of S1P to the cell cultures preferentially stimulated migration of ML1 cancer cells as compared with primary thyroid cells (Figures 3F, G). Most significantly, 20 µM IU1 could block the S1P-mediated increase in ML1 cells, indicating an inhibition of cancer cell migration/invasion. The crystal violet staining of the primary thyroid cells and the ML1 cells that were treated or not are shown in Figures 3F, G **.**

To substantiate these finding we examined FTC-133 representing a more malignant form of thyroid cancer cells. Results obtained showed that IU1 reduced cell proliferation and migration of FTC-133 cells about to the same extent as in ML1 cells (Supplementary Figure S1). Immunoblots cells revealed that the conversion of LC3B was less in the FTC-133 compared with primary thyroid cells (Supplementary Figure S2) which is in line with results obtained with ML1 cells. In contrast to ML1 cells there was no significant reduction of USP14 levels in the FTC-133 cells, suggesting a cell specific regulation of USP14 levels in the two thyroid cancer cell lines.

### Increase in Sphingosine-1-phosphate receptor 3 (S1P3R) in ML1 cancer cells is independent of USP14

**FIGURE 4:**
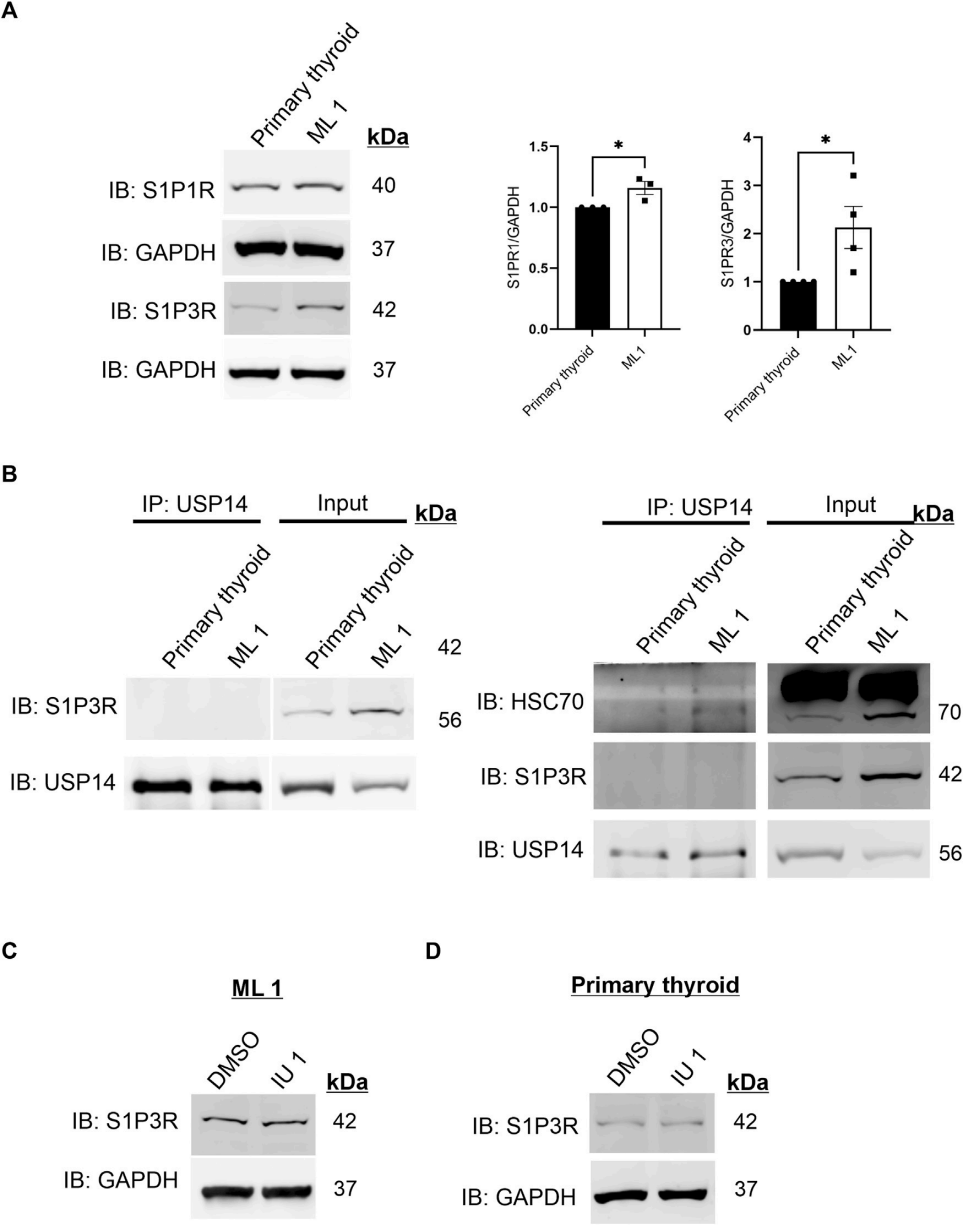
Increases in S1P3 receptor levels in ML1 cells **(A)** Immunoblotting of S1P1R and S1P3R. Right panels represent two histograms of the densitometry ratios of S1P1R and S1P3R, normalized to GAPDH. *n* = 3, *p*-value was calculated by Student’s t-test. *- *p* ≤ 0.05, **- *p* ≤ 0.01, ***- *p* ≤ 0.001. **(B)** Cells lysates from control thyroid and ML1 cells were subjected to immunoprecipitation (IP) using the USP14 antibody. The eluates were immunoblotted for S1P3 receptor (S1P3R) (left) or for HSC70 (right). In the immunoblots, the left panels represent IP and the right ones, the input lysates. **(C,D)** ML1 cells and control thyroid cells were stimulated with 20 µM IU1 for 24 h and subjected to immunoblotting for S1P3 receptor. GAPDH was utilized as a loading control.

S1P is known to bind to the Sphingosine-1-phosphate receptor 1 (S1PR1) and Sphingosine-1-phosphate receptor 3 (S1P3R) on target cells. Previous studies from our laboratory have shown that S1P signaling is important for the migration/invasion of follicular thyroid cancer cells (Kalhori et al., 2013; Kalhori et al., 2016; Asghar et al., 2018). We therefore determined the levels of S1P1R and S1P3R and observed an upregulation of these in the ML1 cancer cells as shown by immunoblotting (Figure 4A). Together these results show that ML1 cells express more receptors for S1P, and that the S1P-induced cell migration can be blocked by using IU1.

Next we investigated the possible interaction of S1P3R with USP14 by performing immunoprecipitation experiments that showed no S1P3R receptors in the immunoprecipitates of USP14 (Figure 4B left). As a positive control, USP14 was able to immunoprecipitate HSC70 under these conditions (Figure 4B right**),** which is in line with our previous data (Srinivasan et al., 2020). Treatment of the cells with IU1 did not reveal any significant changes in S1P3R levels either in ML1 or in the primary thyroid cells (Figures 4C, D). Together these results indicate that there is no interaction between S1P3R and USP14 at least under the present conditions, suggesting that the increase in S1P3R levels occur independently of USP14 or the inhibitor IU1.

### IU1 increases proteasomal activity and changes the composition of 26S proteasomes with less effects on USP14 catalytic activity

**FIGURE 5:**
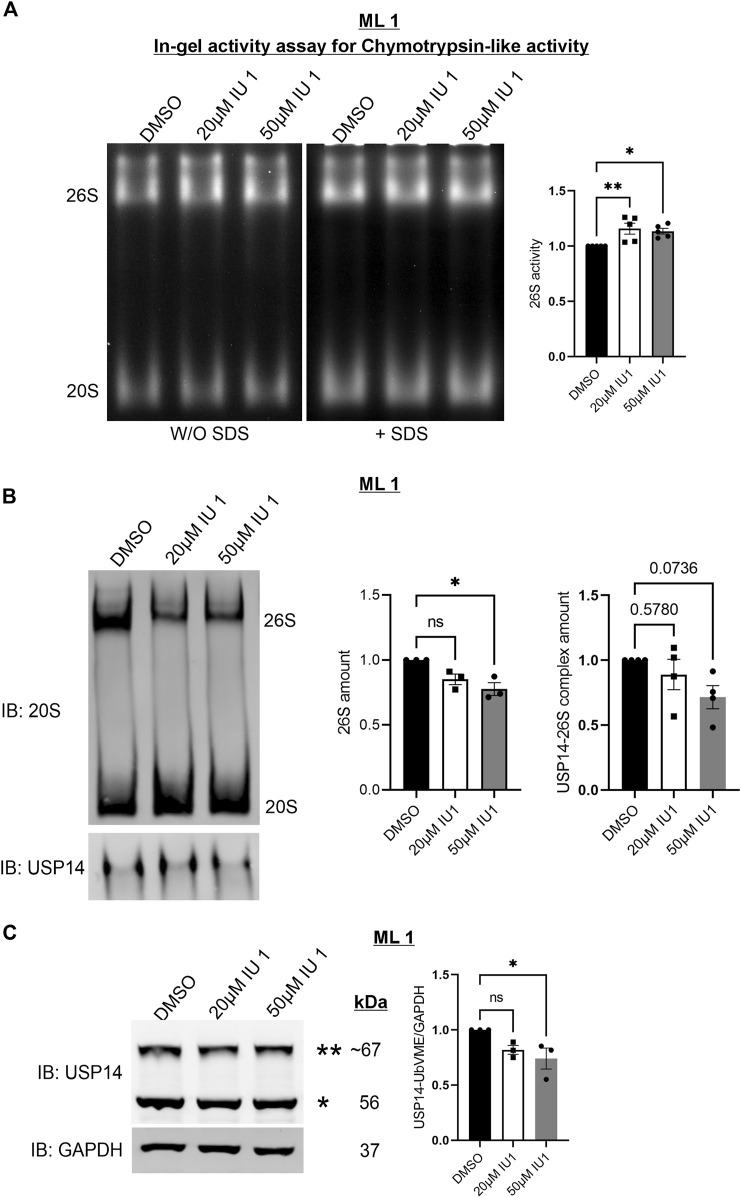
IU1 increases proteasomal activity and changes the composition of 26S proteasomes. ML1 cells were stimulated with 20 µM or 50 µM IU1 for 24 h and analyzed further as indicated below. **(A)** Native-gel electrophoresis followed by in-gel activity assay for chymotrypsin-like activity of the 20S CP of the proteasome. Left panels represent the UV-filter exposed native gel detected with and without the addition of SDS to detect activity of free 20S CP. Right panels represent histogram of the densitometry ratio of 26S proteasome activity in IU1 stimulated samples normalized to the DMSO treated controls. **(B)** Native-gel electrophoresis followed by O/N wet-transfer and immunoblotted with 20S antibody. Right panel panels represent two histograms of the densitometry ratio of 26S and 20S in IU1 stimulated samples normalized to the DMSO treated controls. **(C)** Cell lysates were incubated for 1 h with HA-UbVME substrate at 37°C followed by immunoblotting for USP14 and GAPDH antibodies. Left panel represents the immunoblot of catalytically active USP14 bound to HA-UbVME (**) and free USP14 (*). Right panel represents histogram of the densitometry ration of USP14-UbVME normalized to GAPDH. *n* = 5 **(A)** *n* = 4 **(B)**, *n* = 3 **(C)**, *p*-value was calculated by one-way ANOVA **(B)**. *- *p* ≤ 0.05, **- *p* ≤ 0.01, ***- *p* ≤ 0.001, ****- *p* ≤ 0.0001.

Given the lack of an effect of IU1 on S1P3R levels we next focused on changes by IU1 on protein degradation *via* the 26S proteasomes or autophagy. For this, we analyzed the 26S proteasome activity and composition utilizing native-gel electrophoresis. ML1 cells were stimulated with 20µM and 50 µM IU1 followed by performing an in-gel activity assay (see Methods) with lysates, separated on native gels, for the chymotrypsin-like activity of the 20S core particle (CP) (Figure 5A). Stimulation with IUI increased the catalytic activity of 26S proteasome complex with minor changes in the activity of free 20S CP. Furthermore, we analyzed the amount of the mature 26S proteasomes by immunoblotting using the 20S antibody. Contrary to the above situation, IU1 stimulation decreased the total amount of the 26S proteasomes while showing an increasing trend for the 20S free CP (Figure 5B). These results are in line with the previously known role of IU1 as a proteasome activator (Lee et al., 2010). Furthermore, we noticed that IU1 could reduce the amount of USP14 containing 26S proteasome complexes although the extent of decrease did not reach statistical significance (Figure 5B, lower blots). Further studies are required to reveal the mechanism for activating proteasomes by IU1 and how this can influence the migration properties of the ML1 cells.

To assess if the concentration of 20 µM IU1 could also inhibit the catalytic activity of the USP14 in ML1 cells, we utilized the HA-UbVME substrate binding to the active site. Using this assay, the catalytically active USP14 can be detected in immunoblots as an addition band (Figure 5C). Data obtained showed that treatments with 20 µM IU1 did not significantly influence the activity of USP14 whereas raising the concentration to 50 µM IU1 indeed decreased USP14 activity (Figure 5C). One caution here to consider is the fact that the use of the synthetic substrate HA-UbVME may not fully reflect the situation with natural targets for USP14. However, this observation raised the question whether IU1 could influence some other, catalytic-independent properties of USP14 in the cell.

### IU1 differentially regulates autophagy in ML1 and primary thyroid cells

**FIGURE 6:**
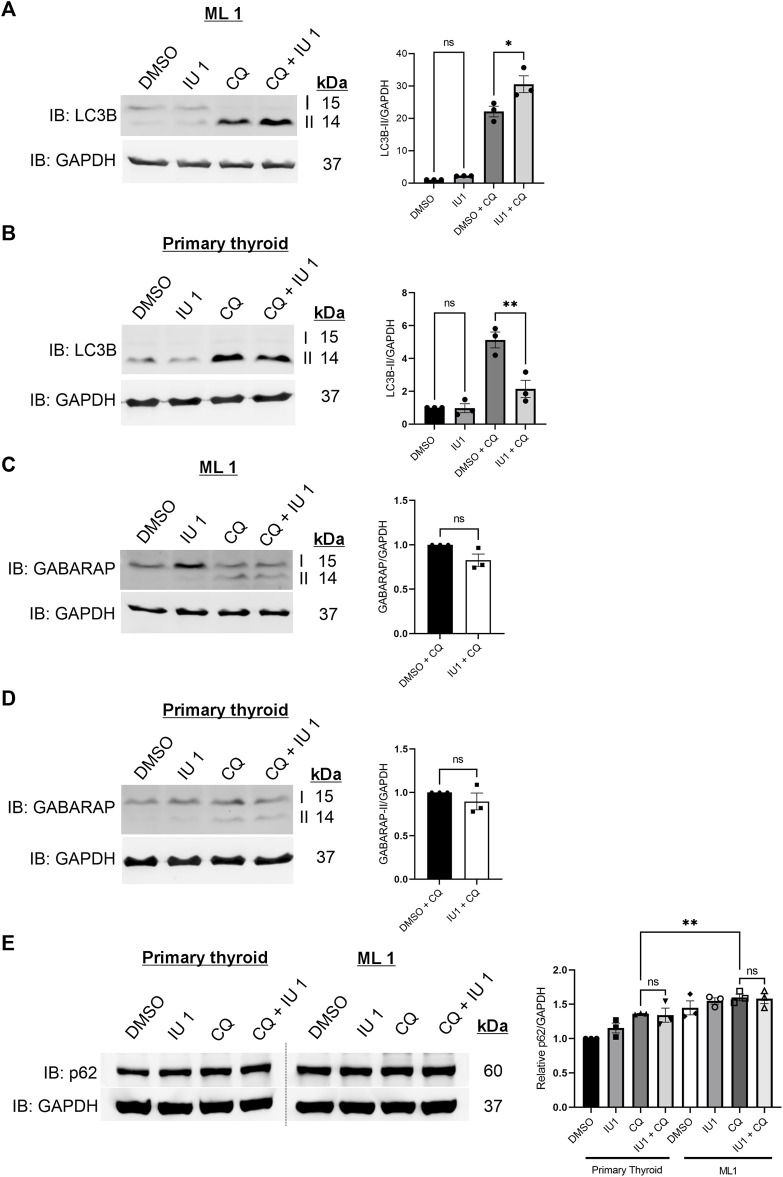
IU1 treatment differentially influences autophagy flux in ML1 and primary thyroid cells with no effect on p62 level. ML1 and primary thyroid cells were stimulated with 20 µM IU1 for 24 h, and 100 uM CQ for 4 h to inhibit the autophagy flux, and cell lysates were subjected to immunoblotting. **(A,B)** Immunoblots LC3B, ML1 cells **(A)**, primary thyroid cells **(B).** Right panels represent histogram of the densitometry ratio of LC3B-II normalized to GAPDH. **(C,D)** Immunoblots GABARAP. ML1 cells **(C)**, primary thyroid cells **(D)**. Right panel represent histogram of the densitometry ratio of GABARAP-II normalized to GAPDH. *n* = 3, *p*-value was calculated by Student’s t-test. *- *p* ≤ 0.05, **- *p* ≤ 0.01, ***- *p* ≤ 0.001. **(E)** ML1 and primary thyroid cells were stimulated with 20 µM IU1 for 24 h, and 100 uM CQ for 4 h to inhibit the autophagy flux, and cell lysates were subjected to immunoblotting for p62 and GAPDH. Right panels represent histogram of the densitometry ratios of p62 and normalized to GAPDH. *n* = 3, *p*-value was calculated by Student’s t-test. *- *p* ≤ 0.05, **- *p* ≤ 0.01, ***- *p* ≤ 0.001.

We next then studied whether IU1 may affect the increased autophagy observed in the ML1 cells (Figure 2). For this, the autophagy flux was analyzed as above using CQ in the absence or presence of 20 µM IU1. Data showed that the addition of IU1 resulted in an accumulation of LC3B-II in ML1 cells (Figure 6A), while primary thyroid cells exhibited a reduced accumulation (Figure 6B). This demonstrates that the IU1 treatment enhances the LC3B-dependent autophagy flux in ML1 cells while reducing it in primary thyroid cells. In contrast, IU1 did not significantly influence GABARAP-dependent autophagy flux neither in ML1 (Figure 6C) nor in primary thyroid cells (Figure 6D).

To study autophagy further, we examined the levels of the protein SQSTM1/p62 that is an autophagy substrate (Klionsky et al., 2016; Liu et al., 2016). We observed that there was an increase in the levels of p62 in ML1 thyroid cancer cells compared with primary thyroid cells (Figure 6E). Treatment with 20 µM IU1 had no significant effect on p62 levels neither in ML1 cells nor in control cells. Addition of chloroquine (CQ) to inhibit autophagic flux increased p62 in both cell lines and the p62 levels were significantly higher in ML1 cells compared with primary thyroid cells (Figure 6E). Taken together the results support the view of an increased autophagy flux in ML1 cells compared with control thyroid cells, however the addition of IU1 did not influence p62 under these conditions.

It was recently reported that the level of the E3 ubiquitin ligase MDM2 is reduced by IU1 in cervical cancer cells along with the activation of autophagy and proteasome (Xu et al., 2020). We were therefore interested to study the levels of MDM2 also in the ML1 thyroid cancer cells as influenced by IU1. Results obtained showed an increase in the basal level of MDM2 in ML1 cells compared with control cells, while IU1 had no significant effect in neither of the cell lines (Supplementary Figure S3). This shows that the regulation of MDM2 is different between various cancer cell types and the effect of IU1 can vary.

In conclusion, we show here that IU1 treatment induces a cell type-dependent response in LC3B-dependent autophagy flux without affecting the GABARAP-dependent autophagy flux. The precise mechanisms by which IU1 differently influences autophagy processes in the ML1, and primary thyroid cells warrant further investigation.

## Discussion

Thyroid cancer is the most common endocrine cancer (Fagin and Wells, 2016). The incidence has steadily increased during the past decades throughout the world. There are different types of thyroid tumors ranging from more benign ones with a relatively good prognosis to malignant anaplastic tumors. Although the follicular type of thyroid cancer is relatively benign some of the tumors can spread and be aggressive warranting the development of novel therapeutic approaches (Nikiforova et al., 2003). In this work, we have shown that the compound IU1 has the capacity to reduce cell proliferation and migration of ML1 follicular and of FTC-133 thyroid cancer cells in culture in a concentration dependent manner. IU1 was more effective in the ML1 cells compared with primary thyroid cells as controls. Likewise, the expression of USP14, being a target for IU1, was reduced in ML1 cells compared with control thyroid cells.

USP14 is a proteasome-associated DUB that plays a multi-faceted role in regulating proteasome activity. USP14 associates with the lid of the proteasomes and is involved in the recognition and trimming of ubiquitin chains on the incoming protein substrate, and in the subsequent activation of the proteasomes (Peth et al., 2009; Lee et al., 2016; Kuo and Goldberg, 2017). Previous studies have shown that USP14 is critical to the cell by maintaining the free ubiquitin pool and by regulation of the proteasome complex (Walters et al., 2008). Recent studies have further suggested that USP14 may play a role in the regulation of ER-stress, and in autophagy by interacting with cellular proteins of these networks (Hyrskyluoto et al., 2014; Srinivasan et al., 2020). A dysregulation of USP14 can alter key cellular processes that in turn can contribute to tumor progression, neurodegenerative pathologies, and inflammation.

Previous studies have suggested that USP14 is a potential target for anti-tumor therapies as shown in cancer cell models of multiple myeloma, lymphomas, breast, lung, liver, and colon cancer (Wang et al., 2016; Abramson, 2018; Jiang et al., 2019; Ma et al., 2020). However, little is so far known about the roles of USP14 in thyroid cancer cell growth and migration. In this work, we have investigated USP14 expression and cell responses towards the inhibitor, IU1 using ML1 cells as an *in-vitro* model for human follicular thyroid cancer. ML1 have previously been used to study thyroid cancer and tumor growth as affected by calcium levels and the bioactive lipid S1P (Asghar et al., 2015).

We first observed that protein levels of USP14 were lower in the ML1 cancer cells in comparison with the control primary thyroid cells. This decrease was not due to the clearance of USP14 by autophagy, which is in contrast to what was reported in another cell type (Sharma et al., 2020). Further, we observed a reduction in USP14 mRNA expression in ML1 cells correlating with the downregulated protein levels. So far, not much is known regarding the transcriptional regulation of USP14 or any mutations in the gene that could affect its expression in different models of cancer. Focusing on the proteasome capacity, there were also no differences in the K48-polyub chains on protein *en route* for proteasomes between ML1 cells and control primary thyroid cells.

Studies of the processes of autophagy in the ML1 cells revealed a lower rate of LC3B and GABARAP conversion compared with control cells, as observed by the amounts of LC3B-II and GABARAP-II in immunoblots (Figure 2). This was also observed in the FTC-133 thyroid cancer cells (Supplementary Figure S2). Likewise, the levels of p62 protein that is an autophagy substrate were slightly increased in the ML-1 cells (Figure 6). These results indicate that autophagy process was altered in the ML-1 cells compared with primary thyroid cells. To study this more, we employed CQ to affect the autophagy flux. Data showed that addition of CQ increased the LC3B- and GABARAP-dependent autophagy flux in the cells and more so in ML1 cells compared with controls. An enhanced autophagy flux is a critical mechanism for regulation of protein turnover and may contribute to tumor cell progression and migration (Folkerts et al., 2019; Chavez-Dominguez et al., 2020; Russell and Guan, 2022). As autophagy is a complex process other steps that autophagy flux likely can also be altered in the ML1 cells compared with control cells, but this will require further studies in the future.

We next focused on the effects of IU1 on cell proliferation, migration as well as the activities of proteasome and autophagy comparing the ML1 cells with correspondingly treated primary thyroid cells. The results obtained show a cell-specific difference between ML1 and control thyroid cells with respect to inhibition of cell viability (MTT assay) and cell proliferation (H_3_-thymidine incorporation) with the tumors cells being more vulnerable towards IU1. This indicates that the ML1 cancer cells are more sensitive to IU1 which could be of benefit for the design of future therapies. It was a bit surprising to note that IU1 more effectively inhibited migration of ML1 cells compared with primary thyroid cells despite the lower levels of USP14 in the ML1 cells (Figure 3G). There is no obvious answer to this apart from the fact that, cancer cells show multiple changes in the regulation of cell invasion and not all these might be amenable to IU1 inhibition. This issue warrants further investigation in the future.

Thyroid cancers particularly of the more aggressive types can spread to other parts of the body or local lymph nodes. For the follicular type of thyroid cancers represented by the ML1 the spreading is usually not to lymph nodes but to organs like bones and lungs. To study migration and effects of IU1, we treated the ML1 cells with 20 µM of the compound that is a concentration not affecting cell proliferation. In these assays, we employed the Transwell chamber both under basal conditions and after stimulation of cells with S1P that is a pro-migratory sphingolipid. Hence as shown here, S1P enhanced cell migration in ML1 cells but not in control primary thyroid cells. This increase was then abrogated by addition of 20 µM IU1 to the cultures. IU1 also inhibited cell migration in ML1 cells as well as in control thyroid cells under basal conditions showing that the compound may influence some common pathways active in both cell types.

To study this, we investigated receptors by which S1P exerts its action on target cells. Previous work from our laboratory has highlighted the importance of S1P3R in cell migration, as evident also here with an increase in pro-migratory S1P3R levels in ML1 cells. Treatment of the cells with IU1 had no effect on the S1P3R levels in ML1 or in control thyroid cells. Furthermore, there was no indication of an interaction between USP14 and S1P3R, as studied by immunoprecipitation experiments. We therefore favor the view that the inhibition of cell migration upon treatment with 20 µM IU1 could be due to other signaling mechanisms than S1P receptors.

Previous studies have established a complex context-dependent role for autophagy and proteasome in the regulation of tumor cell progression. The proteasome and autophagy are involved in the protein clearance of substrates that affect cell processes critical for tumor growth and invasion.

Thus, targeting the proteasome could provide an important anti-tumor strategy in various cancer types (D'Arcy et al., 2011; Huang et al., 2017; Morozov and Karpov, 2019; Chavez-Dominguez et al., 2020). Therefore, we focused here on changes in proteasome activity in ML1 cells upon treatment with IU1. Native-gel in-gel activity assays showed that IU1 treatment increased the chymotrypsin-like activity of the 26S proteasomes with no effects on the free 20S CP. Interestingly, whilst the activity was increased, the protein amount of the mature 26S proteasome complex was downregulated upon treatment with IU1. Previous studies using breast cancer cells have shown that the activation of proteasomes can reduce cell migration and proliferation (Miettinen et al., 2018). This is accordance with data showing that IU1 can activate the proteasome.

Studying the catalytic activity of USP14 using the synthetic substrate HA- UbVME and 20 µM IU1 revealed no significant inhibition of USP14, whereas addition of 50 µM IU1 had an effect. One caution here is the fact that the use of the synthetic substrate may not fully reflect the situation with natural targets for USP14. However, this observation also raised the question whether IU1 could influence some proteasome-independent functions of USP14 in the cell like autophagy. Results showed that the autophagy flux was enhanced in ML1 cells but inhibited in control thyroid cells by using IU1. With respect to GABARAP-dependent flux, there were no significant changes in neither ML1 nor in control thyroid cells. These results suggest that IU1 influences mainly the LC3B-dependent autophagic flux in the ML1 cells. It has been considered that LC3B and GABARAP family of ATG8s may serve specific roles at distinct stages during autophagy, with GABARAPs potentially involved in the later stages of autophagosome maturation and fusion. Recent findings have indicated a possibly role for GABARAPs in selective autophagy such as parkin-dependent mitophagy (Weidberg et al., 2010; Wang et al., 2015; Nguyen et al., 2016; Jacquet et al., 2021; Reid et al., 2022). It would be important to assess the lipidation and accumulation of both LC3B and GABARAP upon autophagy inhibition to increase our understanding of their respective roles in various tumors.

In summary, in this work we have identified novel mechanisms of how IU1 acts in tumor cells using ML1 thyroid cancer cells as a model. IU1 was found to dramatically affect the cell proliferation of the ML1 cells compared with control primary thyroid cells. IU1 further significantly affected the proteasome activity, and LC3B-dependent autophagy flux in the ML1 cells. Further studies are required to characterize specifically the substrates that are involved in regulating proliferation and migration as well as autophagy and proteasomes in ML1 cells. In addition, we observed that the protein and mRNA levels of USP14 were lower in ML1 cells compared with control primary thyroid cells. This was however not observed with FTC-133 cells indicating that the USP14 levels may vary between different thyroid cancer cells, which will require further investigations. Taken together the present findings show novel roles of IU1 in targeting thyroid cancer cells that could be applicable to other tumor cells as well.

## Data Availability

The original contributions presented in the study are included in the article/Supplementary Material, further inquiries can be directed to the corresponding author.

## Supplementary material

SUPPLEMENTARY FIGURE S1 IU1 inhibits the proliferation and S1P-evoked migration of FTC-133 follicular thyroid cancer cells. **(A)** FTC-133 thyroid cancer cells were stimulated with DMSO, Mitomycin C or IU1 1-100 µM in the presence of radioactive thymidine as described in Methods. Cell proliferation was significantly decreased on higher concentrations of IU1. Values are means ± S.E.M, *n* = 3. ***p* < 0.01, ****p* < 0.001. **(B)** Cell invasion of FTC-133 thyroid cancer cells were investigated in the absence or presence of 20 µM IU1 or S1P 100 nM. IU1 decreased basal invasion and had no further effect on S1P evoked invasion. Values are means ± S.E.M, *n* = 3. ****p* < 0.001, *****p* < 0.0001. Representative images of cells in Transwell inserts and stained with crystal violet stain are shown to the right. The scale bar is 50 µm.

SUPPLEMENTARY FIGURE S2 Immunoblots of FTC-133 thyroid cancer cells Primary thyroid cells and FTC133 cells were cultured and analyzed as below. Upper panels. Left, Immunoblotting for USP14. Right panel, histogram of the densitometry ratio of USP14 normalized to GAPDH. Lower panels. Left, Immunoblotting for LC3B. Right panel, histogram of the densitometry ratio of LC3B-II normalized to LC3B-I as a measure of LC3B conversion. *n* = 3,Values are mean ± S.E.M. p-value was calculated by Student’s t-test. *- *p* ≤ 0.05, **- *p* ≤ 0.01, ***- *p* ≤ 0.001, *****p* < 0.0001.

SUPPLEMENTARY FIGURE S3 MDM2 expression in ML-1 and control thyroid cells ML1 and primary thyroid cells were stimulated with 20 µM IU1 for 24 h and cell lysates were subjected to immunoblotting for MDM2 and GAPDH. Right panels represent histogram of the densitometry ratios of MDM2 and normalized to GAPDH. *n* = 3, p-value was calculated by Student’s t-test. *- *p* ≤ 0.05, **- *p* ≤ 0.01, ***- *p* ≤ 0.001.

SUPPLEMENTARY FIGURE S4 Full blots for all immunoblots represented in the manuscript along with the molecular weight ladder.
