# Supplementary figures and images for: Proliferation and migration of ML1 follicular thyroid cancer cells are inhibited by IU1 targeting USP14: role of proteasome and autophagy flux
Source: Front Cell Dev Biol. 2023 Aug 30;11:1234204. doi: 10.3389/fcell.2023.1234204 (PMC10499180; doi:10.3389/fcell.2023.1234204)

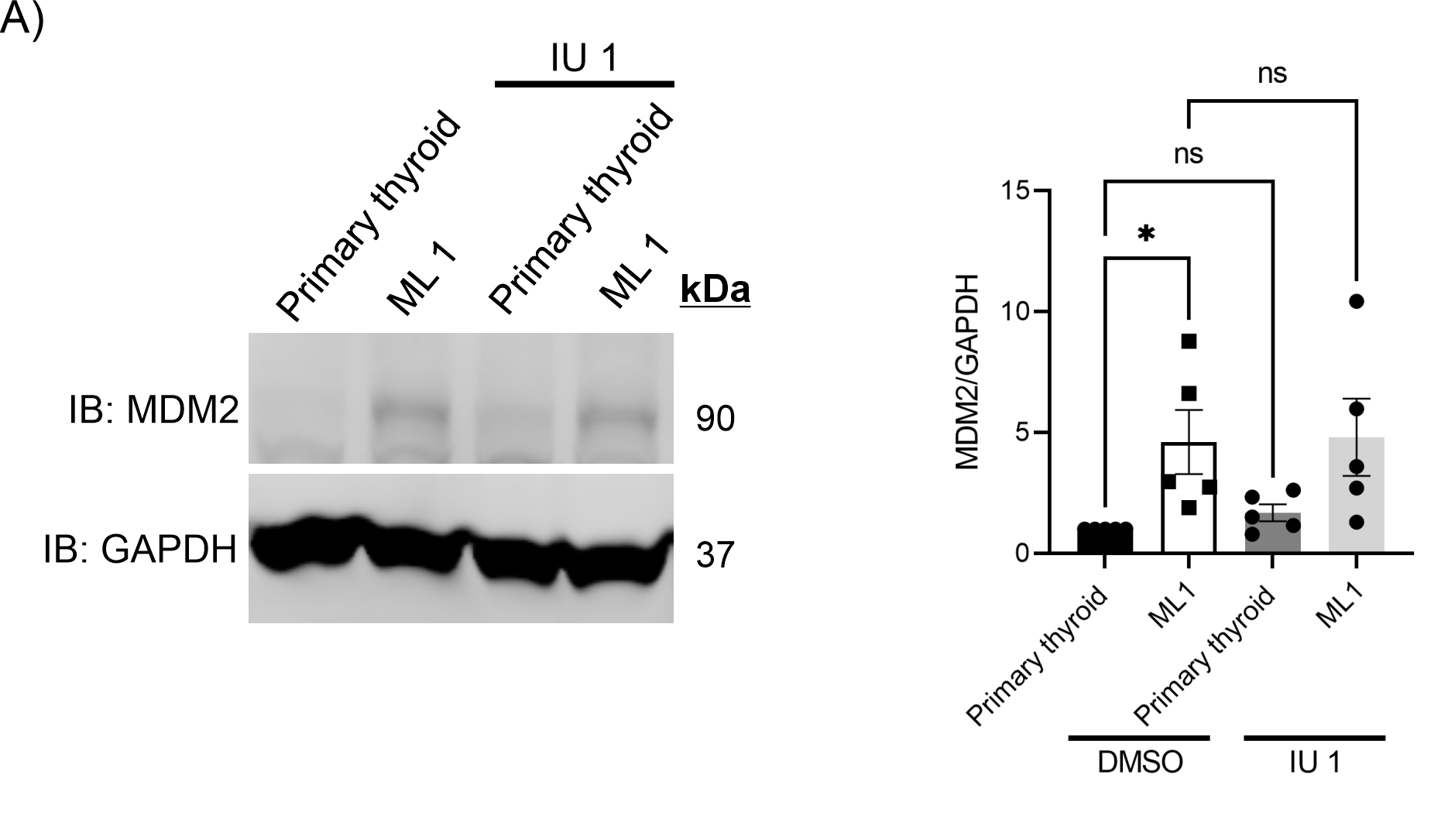

Supplement: Supplementary file 1 [file Image3.TIF]

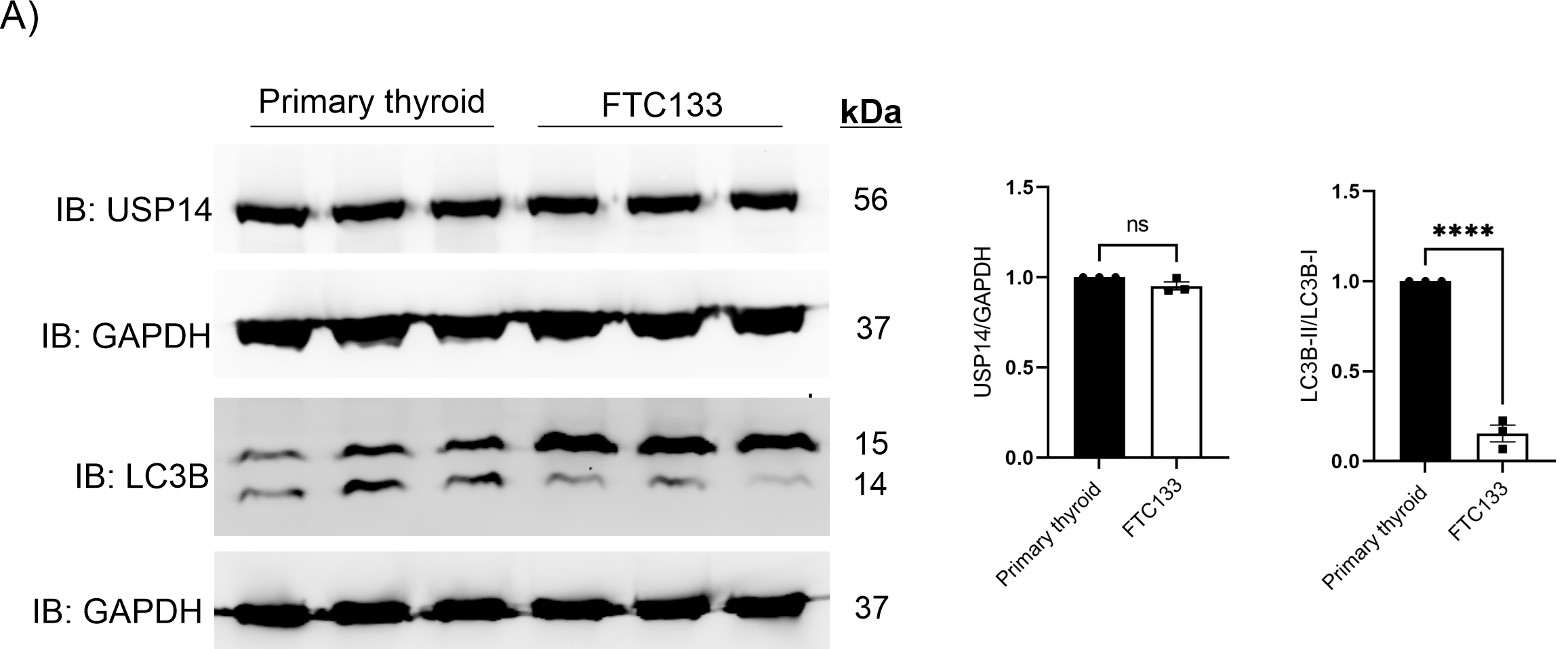

Supplement: Supplementary file 2 [file Image2.TIF]

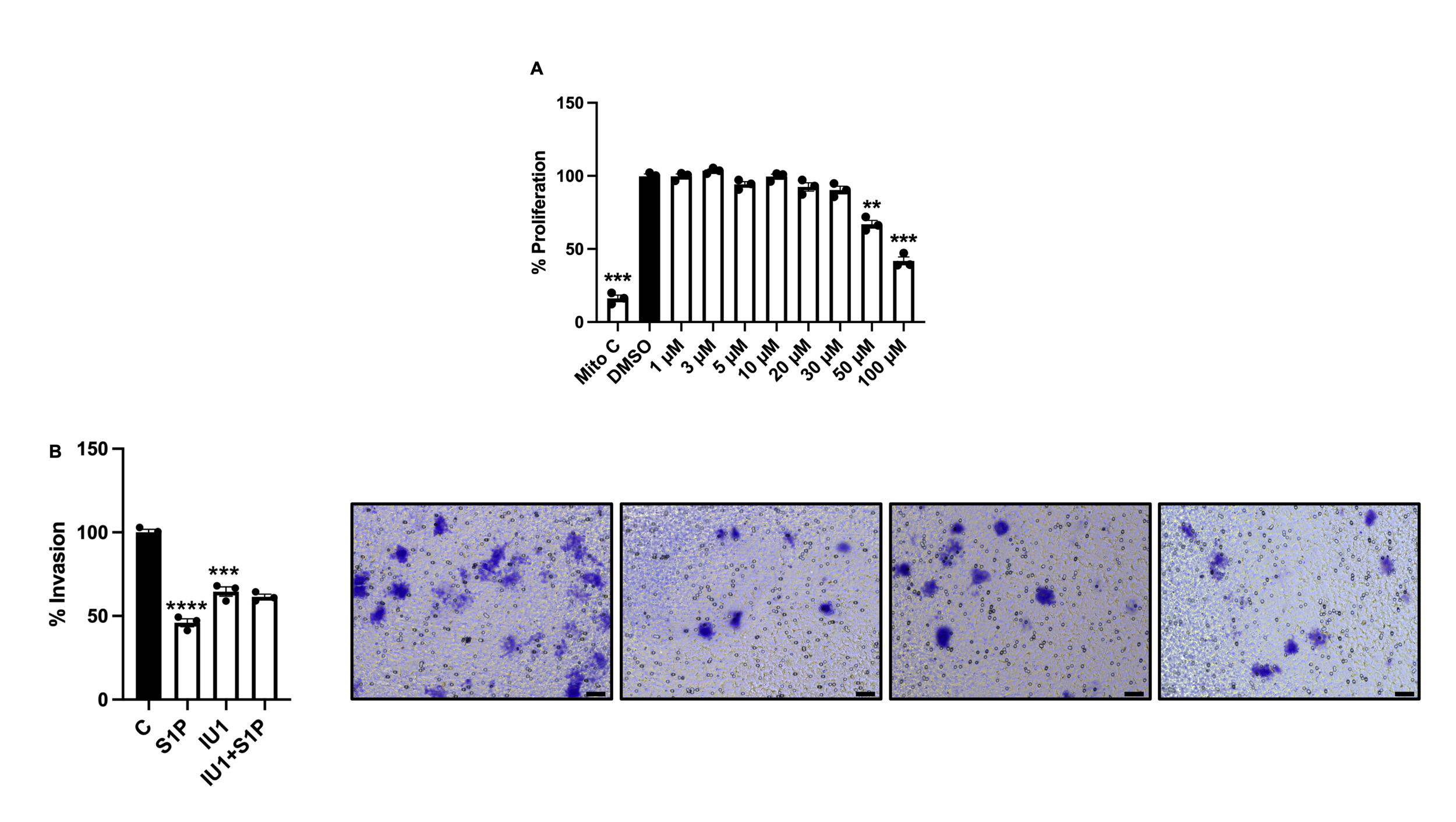

Supplement: Supplementary file 3 [file Image1.TIF]
