# Supplementary material for: Proliferation and migration of ML1 follicular thyroid cancer cells are inhibited by IU1 targeting USP14: role of proteasome and autophagy flux
Source: Front Cell Dev Biol. 2023 Aug 30;11:1234204. doi: 10.3389/fcell.2023.1234204 (PMC10499180; doi:10.3389/fcell.2023.1234204)
Supplement: Supplementary file 4 [file DataSheet1.PDF]

Figure 1

Protein molecular weight ladder used: PageRuler Plus Prestained protein ladder

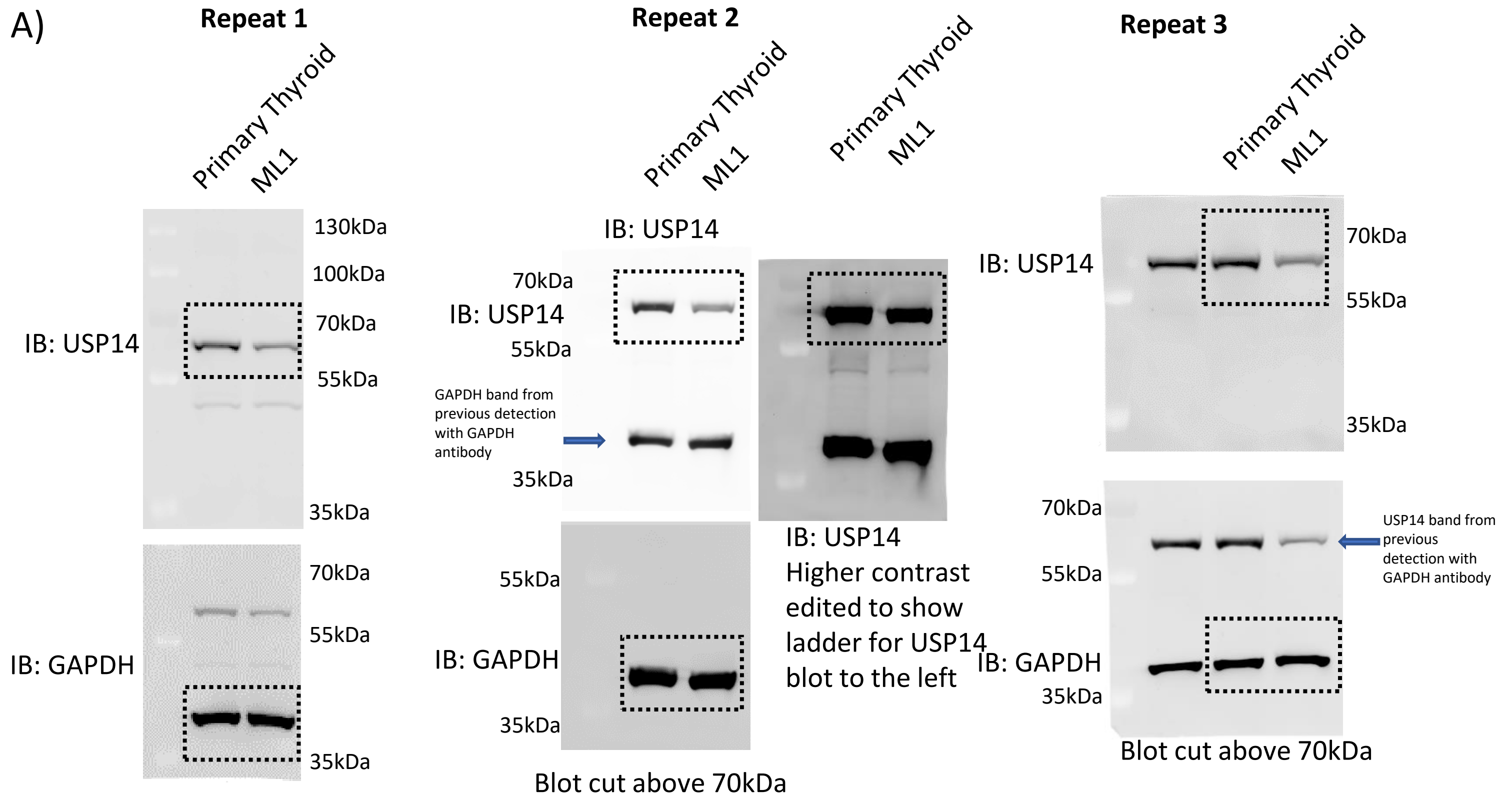

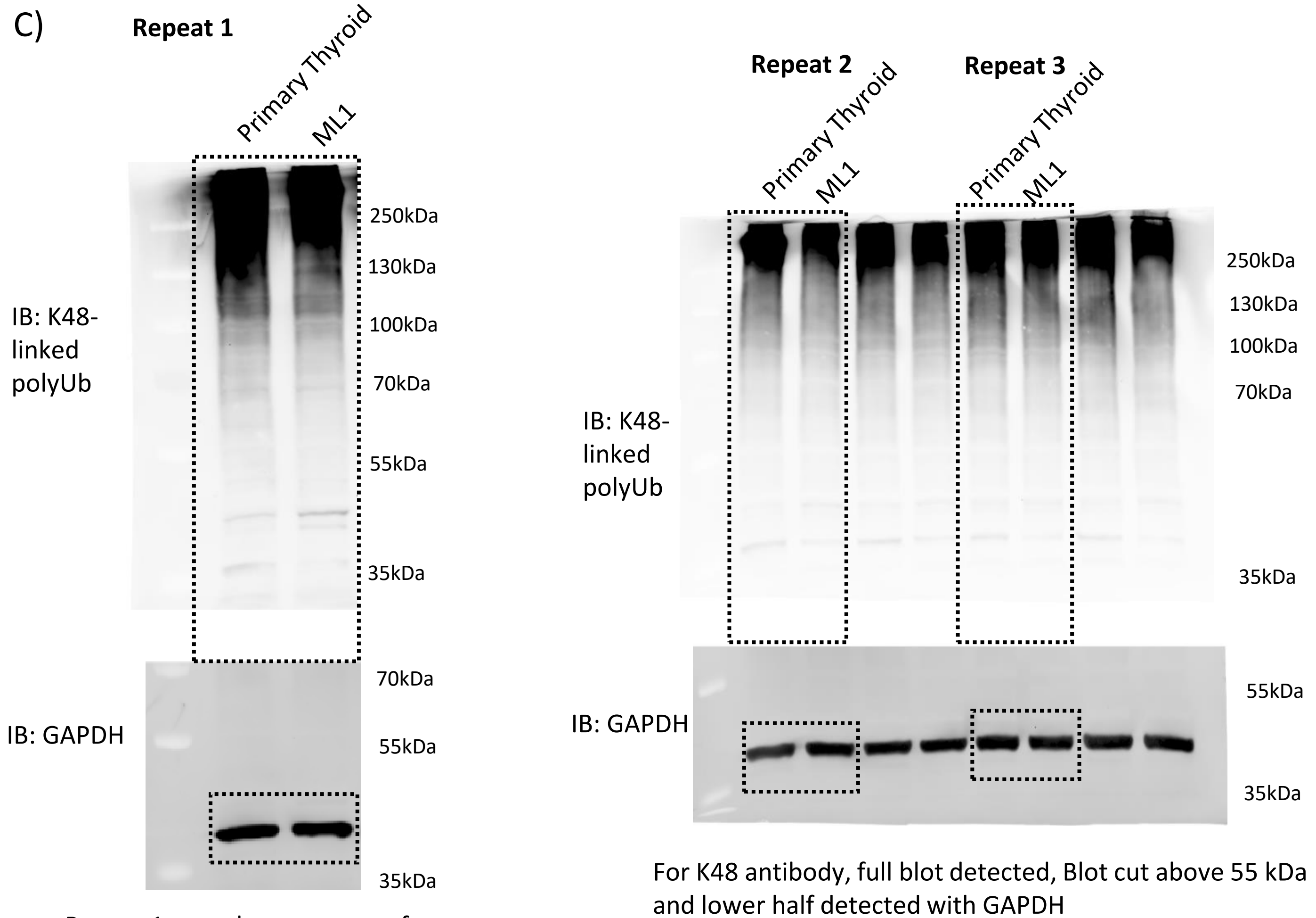

Repeat 1, membrane not cut, for GAPDH antibody, the scanner was imaged from the 70kDa marker to lower end of membrane

For K48 antibody, full blot detected, Blot cut above 55 kDa and lower half detected with GAPDH
Repeats 2 and 3 were run on the same gel and are indicated in dotted rectangular box. The other 4 lanes are samples that have not been included in the manuscript

Figure 2

A)

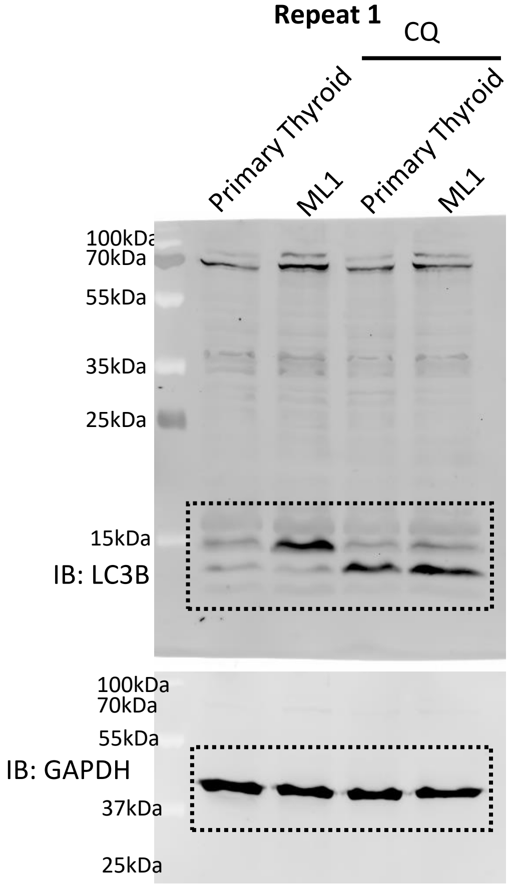

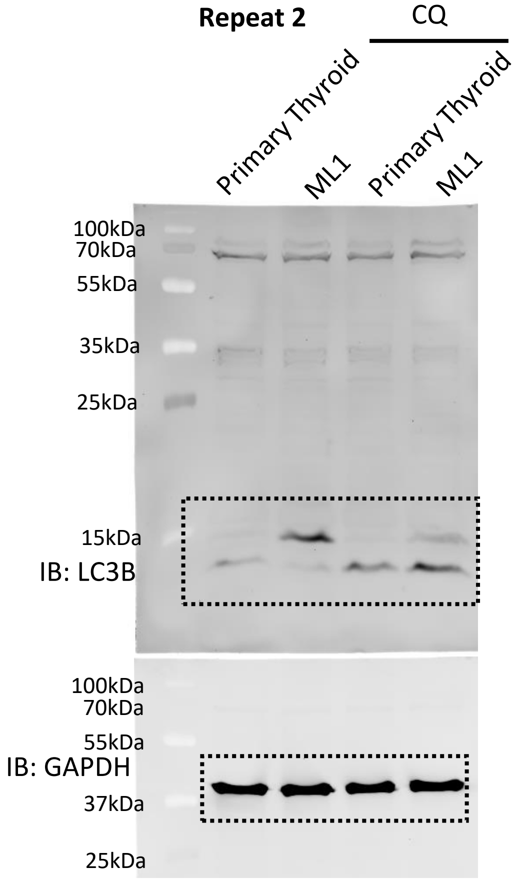

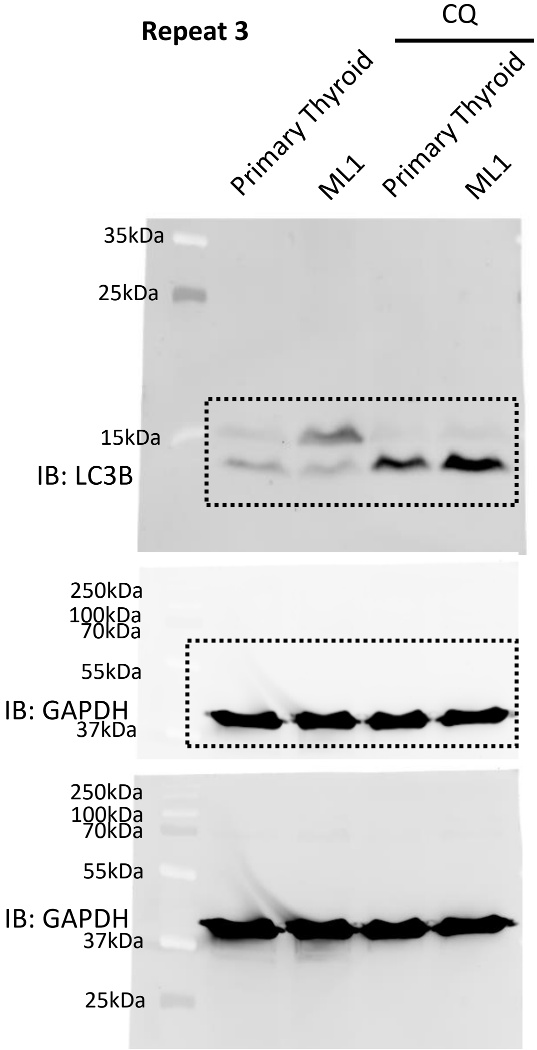

Repeats 1 and 2, blots were not cut. During imaging, imaging area for LC3B antibody was from the 100kDa to the lower end of membrane

Repeat 3, Blot was not cut. During imaging,for LC3B antibody, the imaging area on the imager was selected only from 35kDa to the lower end of membrane.

All the 3 repeats For GAPDH, The imaging area on scanner was from the 25kDa to the upper end of the membrane

GAPDH blot same as above
Higher contrast edited to show ladder

Protein molecular weight ladder used: PageRuler Plus Prestained protein ladder

Figure 2

Protein molecular weight ladder used: PageRuler Plus Prestained protein ladder

B)

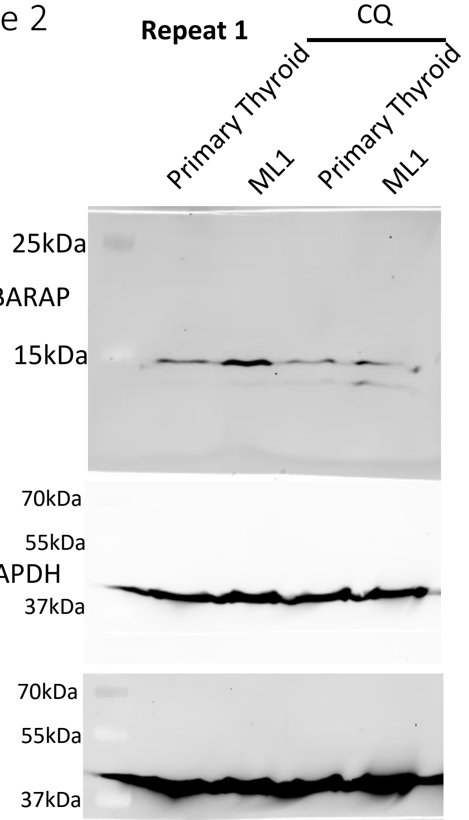

GAPDH blot seen above, contrast edited to show the MW ladder clearly.

Repeat 1, membrane was cut at above 25kDa to detect GAPDH, lower part with GABARAP

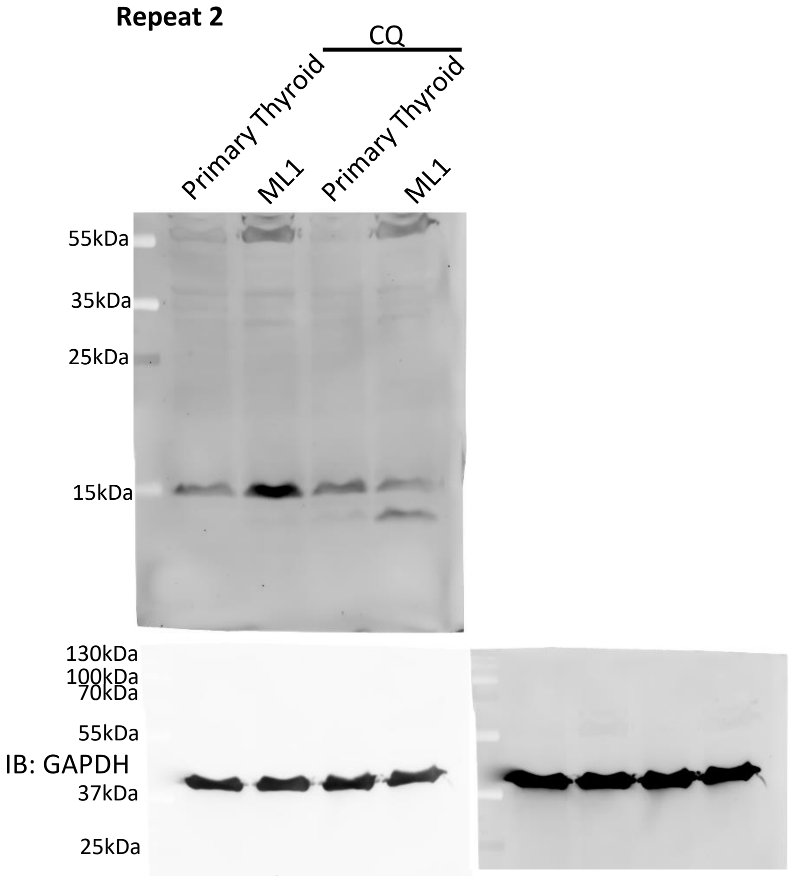

IB: GAPDH

IB: GAPDH seen to the left, contrast edited to show the MW ladder clearly.

Repeat 2 membrane not cut. but during imaging, for GABARAP antibody, the imaging area on the imager was selected only from 55kDa to the lower end of membrane. For GAPDH antibody, imaging area was between 130kDa to 25kDa

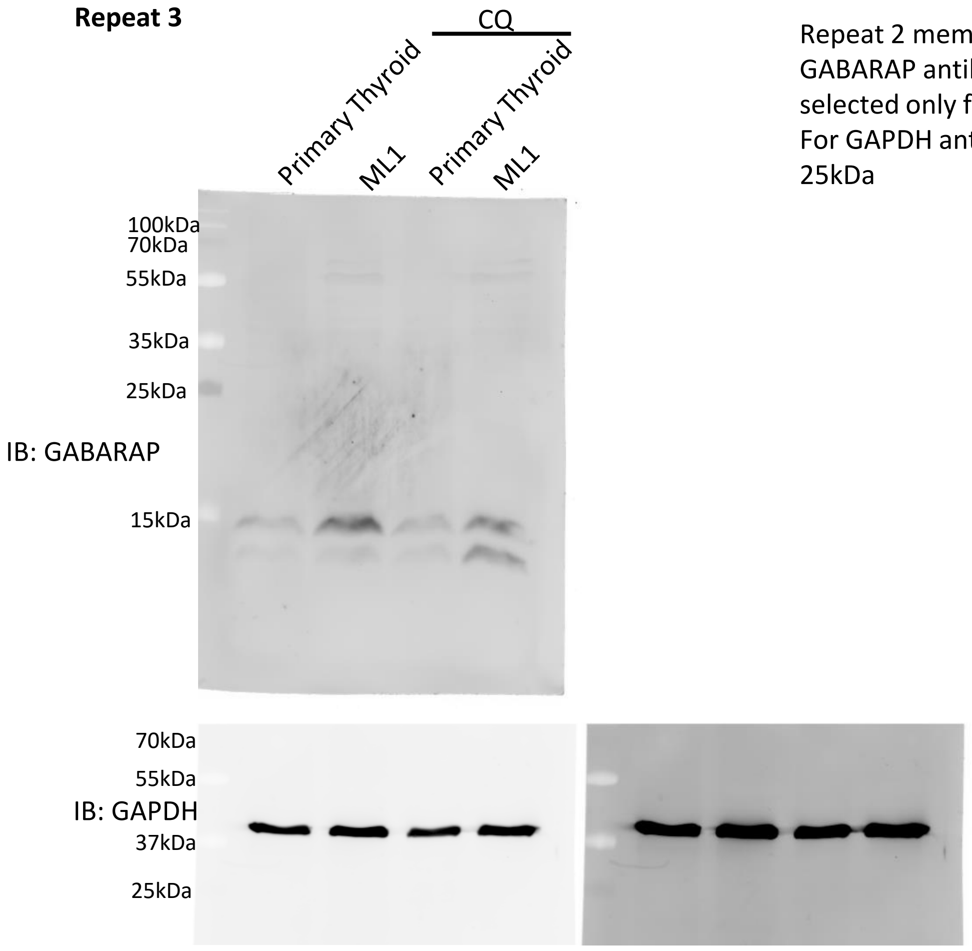

IB: GAPDH seen to the left, contrast edited to show the MW ladder clearly.

Repeat 3 membrane not cut. but during imaging, for GABARAP antibody, the imaging area on in the imager was selected only from 100kDa to the lower end of membrane.
For GAPDH antibody, imaging area between 70kDa to below the 25kDa MW marker

Figure 2

Protein molecular weight ladder used: PageRuler Plus Prestained protein ladder

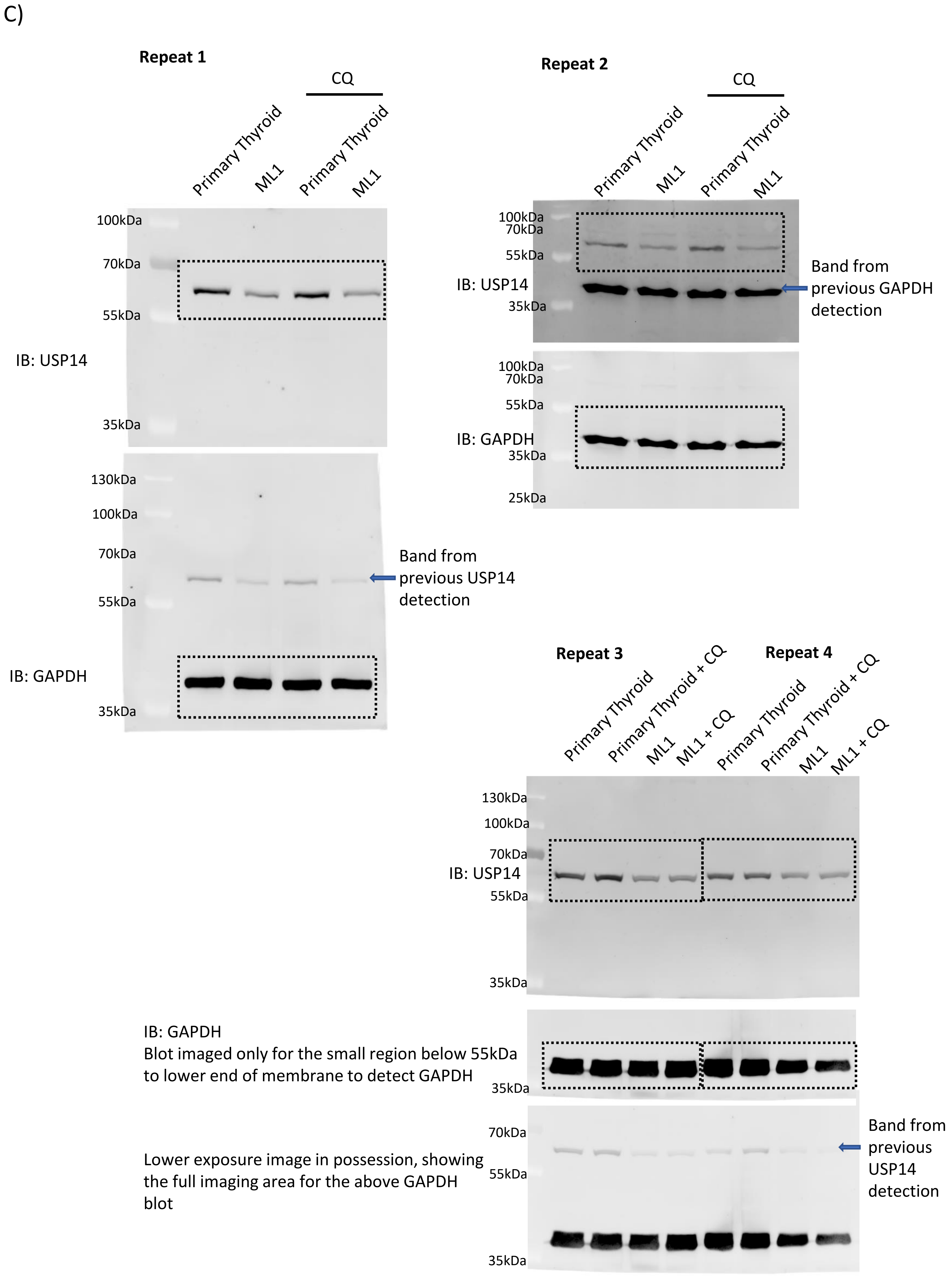

Figure 4

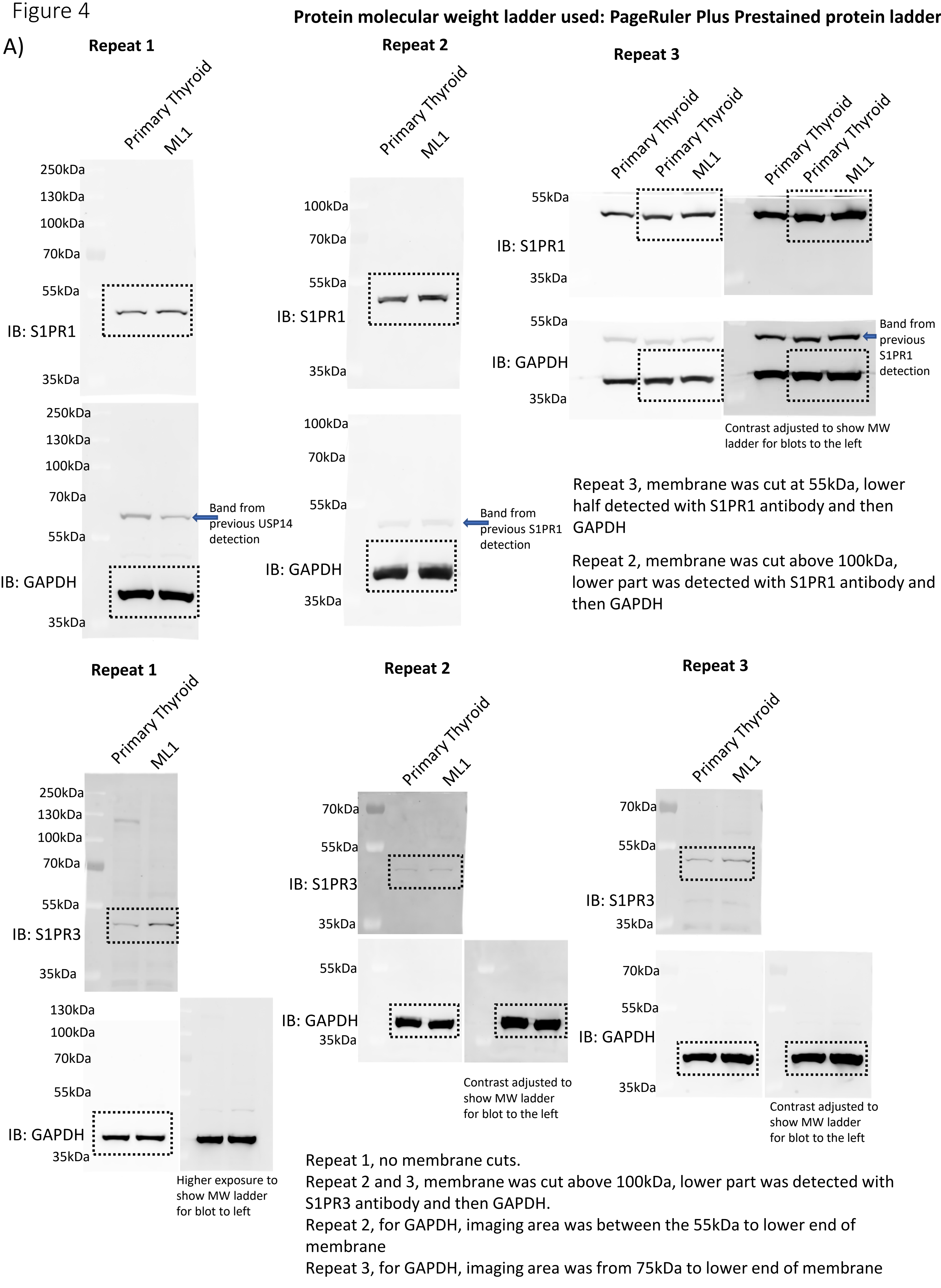

Protein molecular weight ladder used: PageRuler Plus Prestained protein ladder
A)
Repeat 1
Primary Thyroid
ML1
250kDa
130kDa
100kDa
70kDa
55kDa
IB: S1PR1
35kDa
IB: GAPDH
Band from previous USP14 detection
Repeat 2
100kDa
70kDa
55kDa
35kDa
IB: S1PR1
IB: GAPDH
Band from previous S1PR1 detection
Repeat 3
IB: S1PR1
IB: GAPDH
Band from previous S1PR1 detection
Contrast adjusted to show MW ladder for blots to the left
Repeat 3, membrane was cut at 55kDa, lower half detected with S1PR1 antibody and then GAPDH
Repeat 2, membrane was cut above 100kDa, lower part was detected with S1PR1 antibody and then GAPDH
IB: S1PR3
Higher exposure to show MW ladder for blot to left
Contrast adjusted to show MW ladder for blot to the left
Repeat 1, no membrane cuts.
Repeat 2 and 3, membrane was cut above 100kDa, lower part was detected with S1PR3 antibody and then GAPDH.
Repeat 2, for GAPDH, imaging area was between the 55kDa to lower end of membrane
Repeat 3, for GAPDH, imaging area was from 75kDa to lower end of membrane

Figure 4

Protein molecular weight ladder used: PageRuler Plus Prestained protein ladder

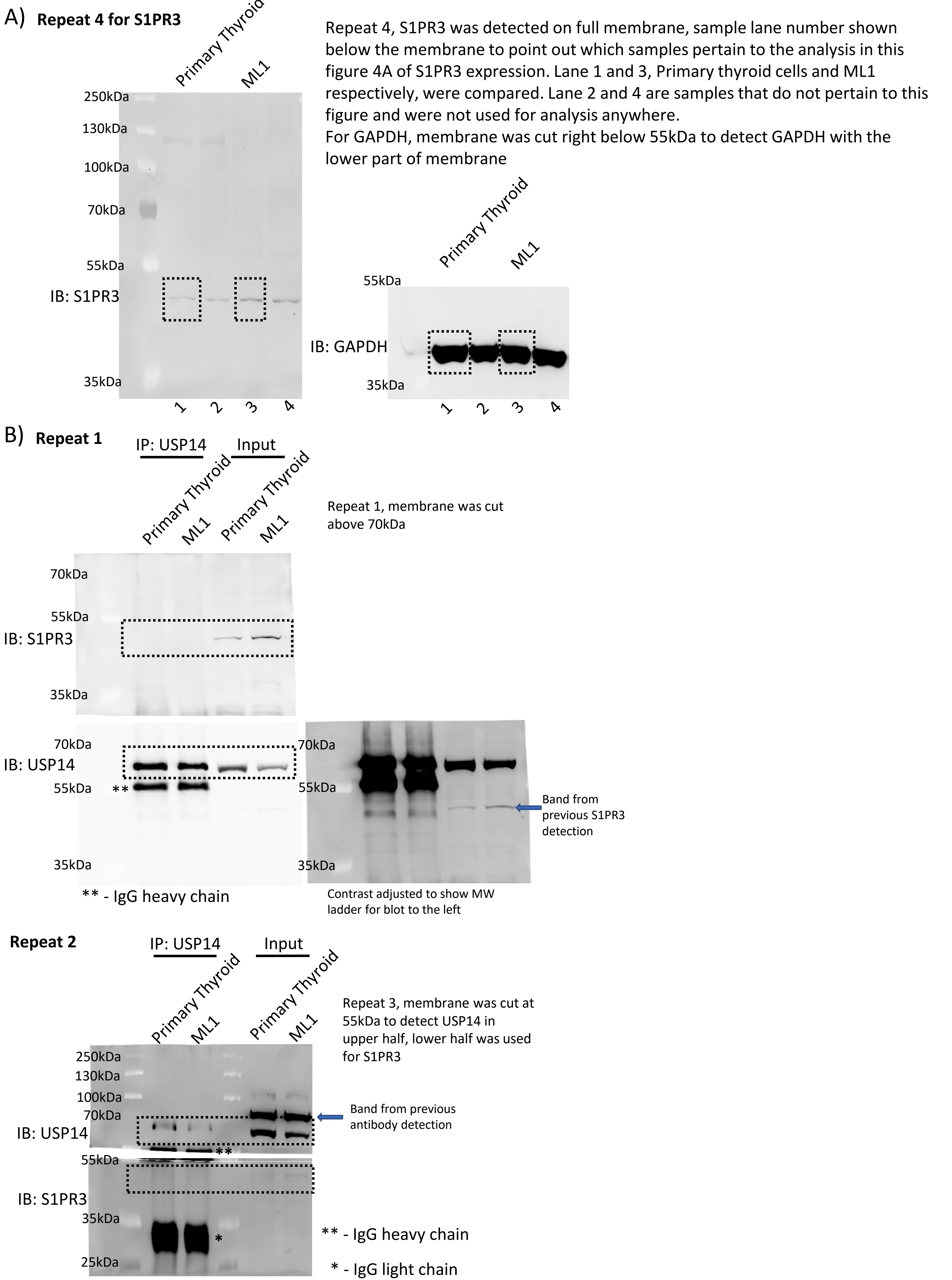

Figure 4

Protein molecular weight ladder used: PageRuler Plus Prestained protein ladder

B)

Repeat 3 and full blot of the figure 4B right panel

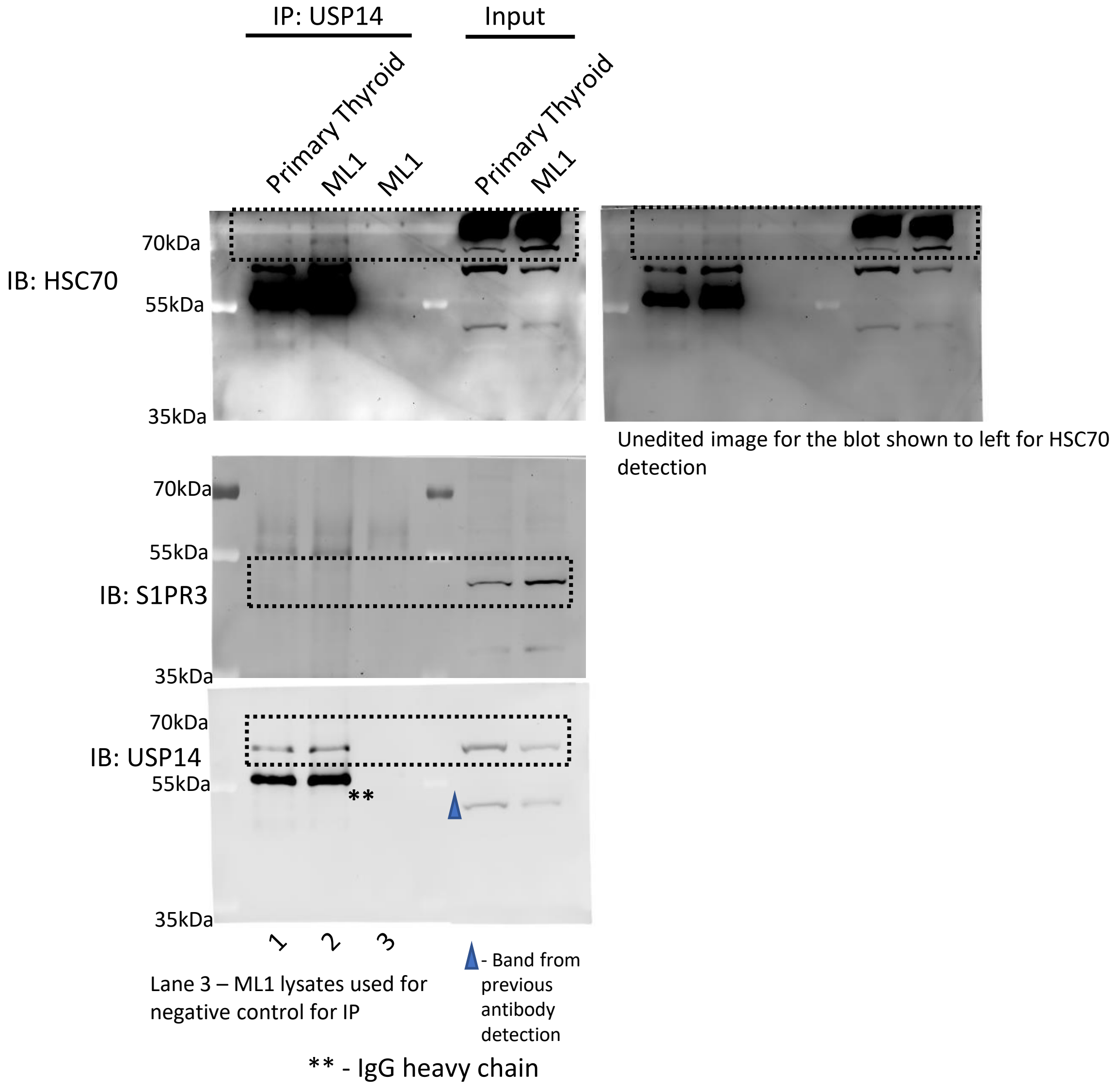

membrane was cut below 100kDa and lower part was detected with HSC70

Figure 4

C)

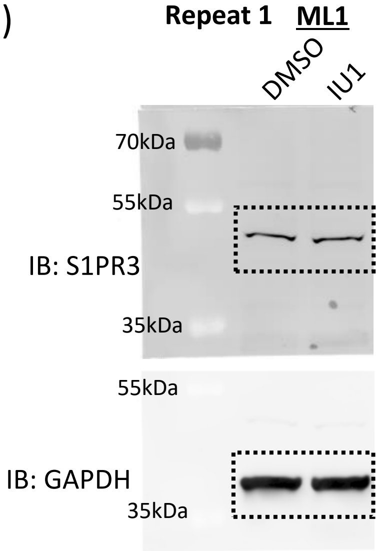

Repeat 1, membrane was cut above 70kDa. For GAPDH, membrane detected from 55kDa to lower end of membrane

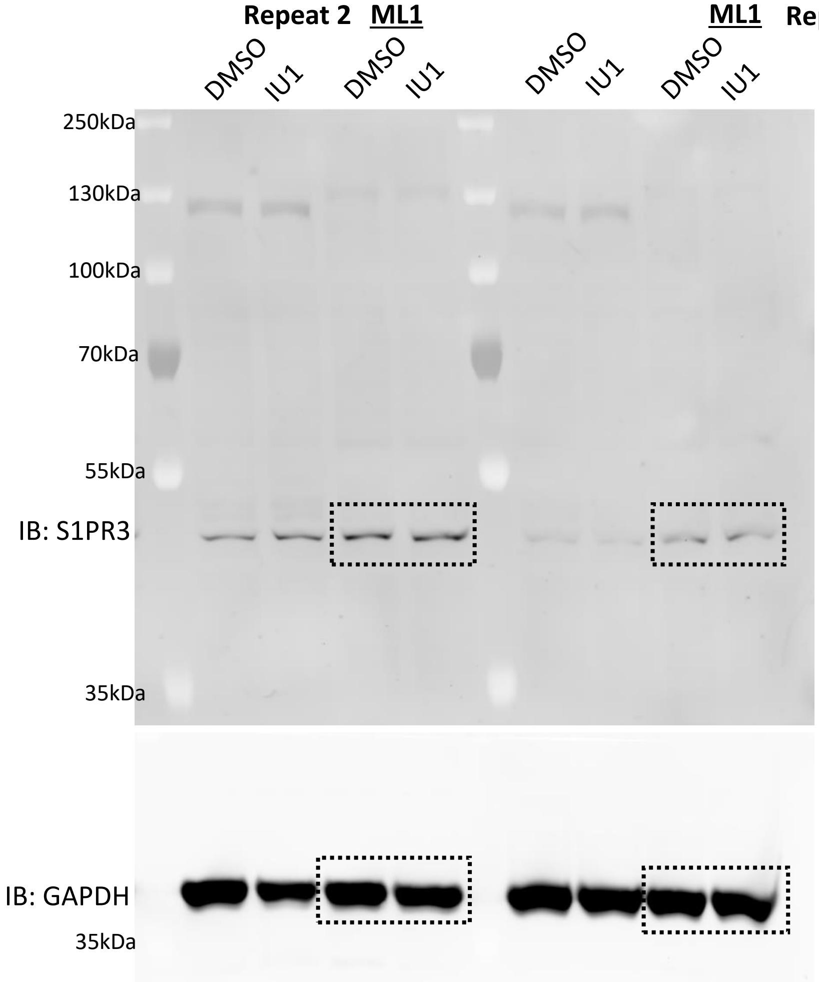

Repeat 2 and 3, run on the same gel, sample lanes have been marked above the membrane for each lane.

For GAPDH, membrane was cut right below 55kDa. Lower part was used for detecting GAPDH

D)

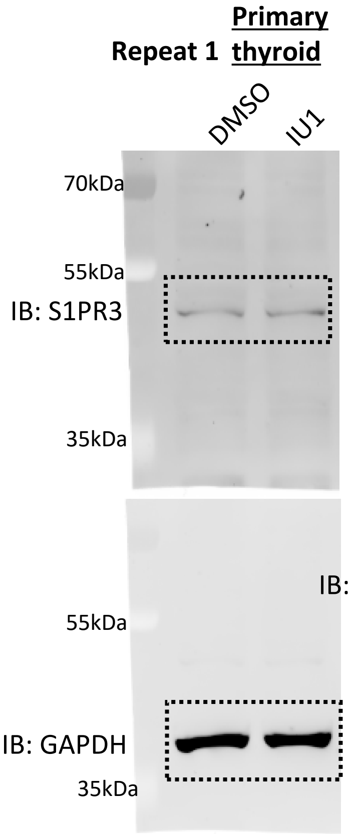

Repeat 1, membrane was cut above 70kDa, and the lower half was detected with S1PR3 and then GAPDH

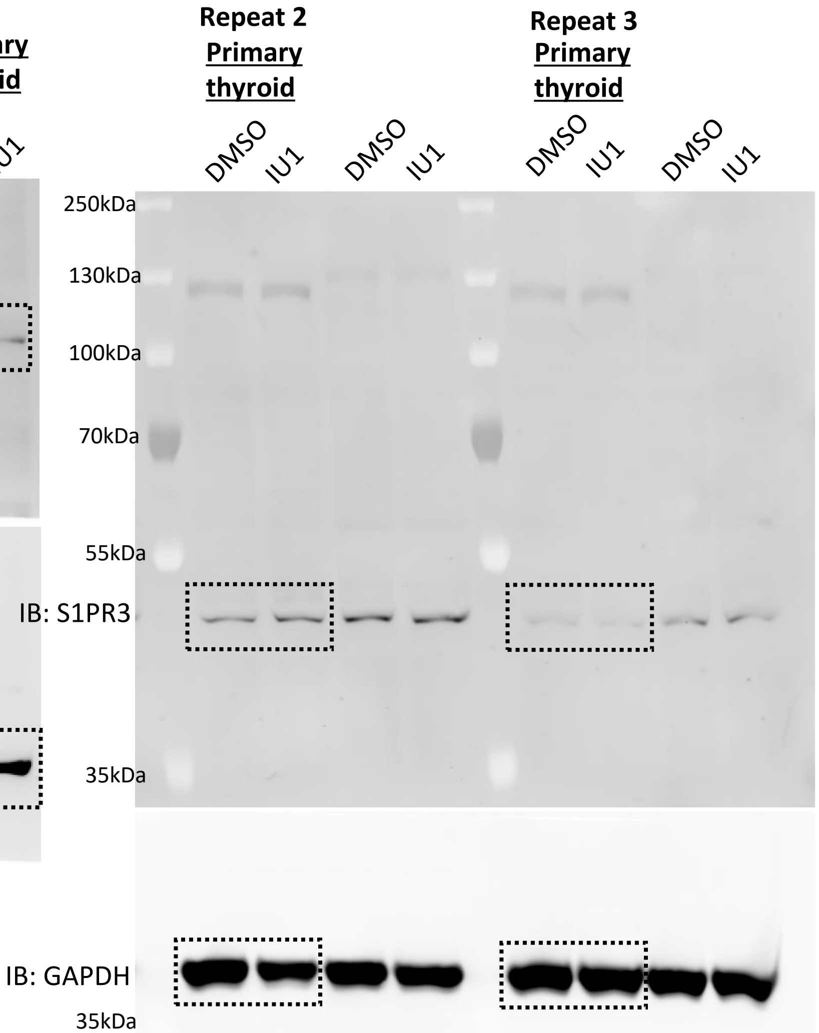

Repeat 2 and 3, run on the same gel, sample lanes have been marked above the membrane for each lane.

For GAPDH, membrane was cut right below 55kDa. Lower part was used for detecting GAPDH

Protein molecular weight ladder used: PageRuler Plus Prestained protein ladder

Figure 5

A)

Repeat 1

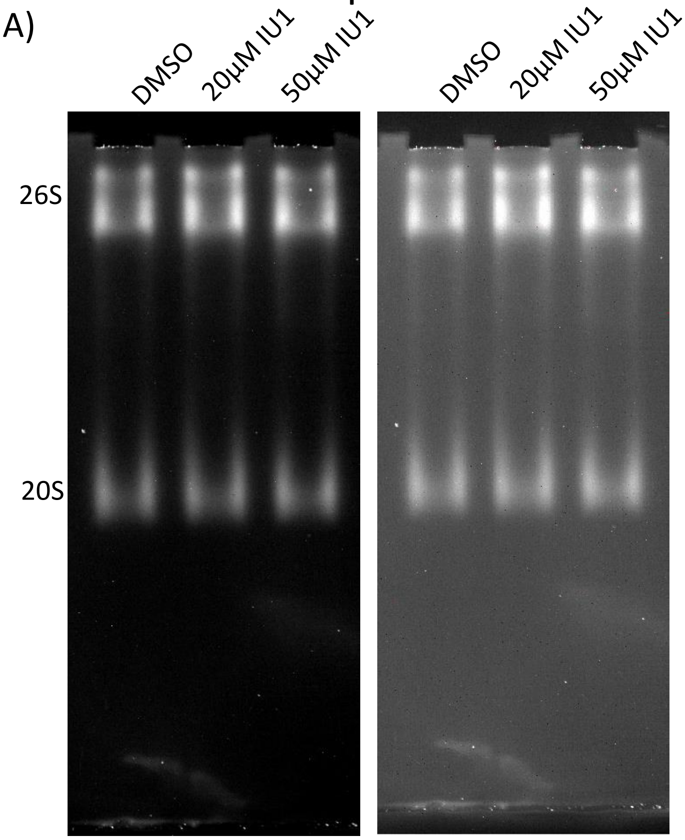

Representative figure 4A full gel
26S activity seen here not used for quantification.
See to the right, the unprocessed gel image that was utilized fo

Unprocessed/unedited image of gel from figure 4A used for quantification of 26S activity

Repeat 2

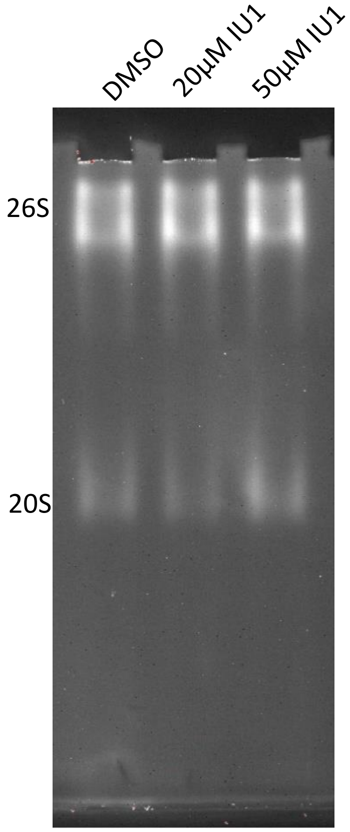

Repeat 3

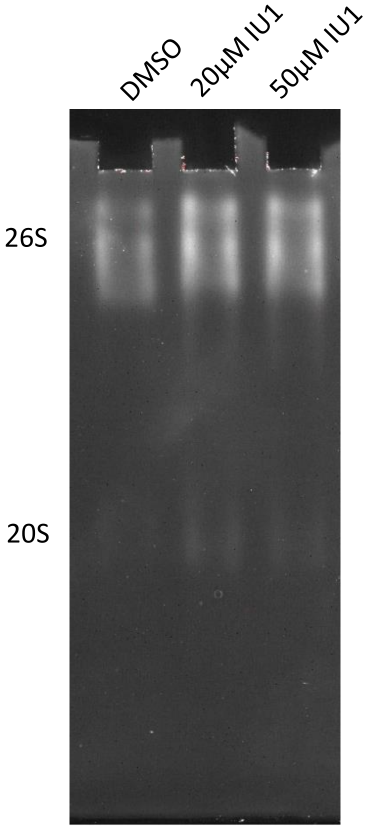

No protein ladder in native gels

Repeat 4

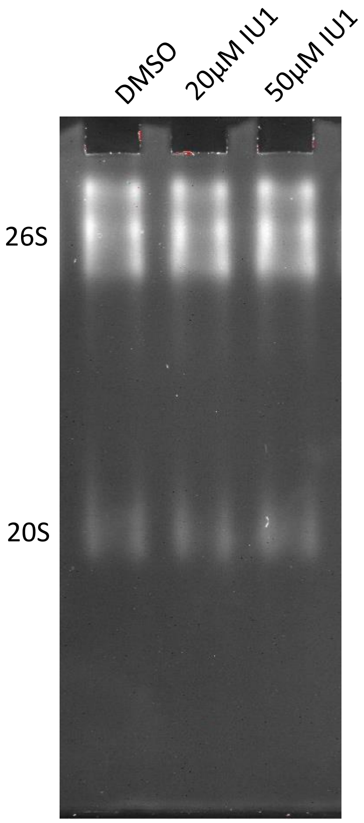

Repeat 5

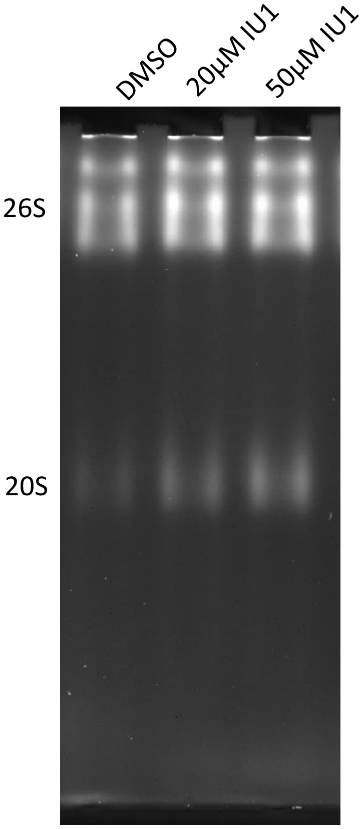

Figure 5

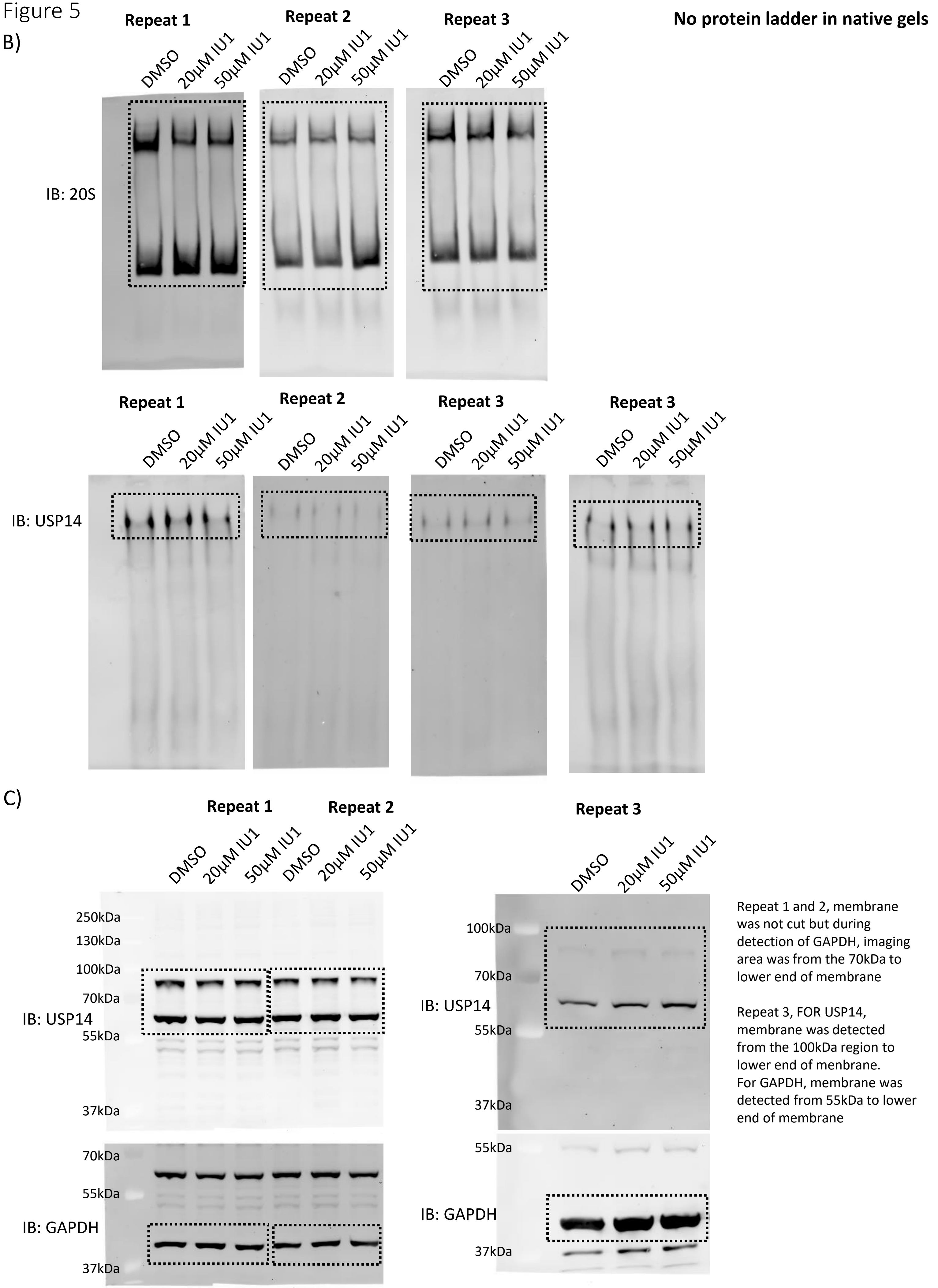

B)
Repeat 1
Repeat 2
Repeat 3
No protein ladder in native gels
DMSO
20µM IU1
50µM IU1
IB: 20S
Repeat 1
Repeat 2
Repeat 3
Repeat 3
IB: USP14
C)
Repeat 1
Repeat 2
Repeat 3
250kDa
130kDa
100kDa
70kDa
55kDa
37kDa
IB: USP14
IB: GAPDH
Repeat 1 and 2, membrane was not cut but during detection of GAPDH, imaging area was from the 70kDa to lower end of membrane
Repeat 3, FOR USP14, membrane was detected from the 100kDa region to lower end of menbrane.
For GAPDH, membrane was detected from 55kDa to lower end of membrane

Figure 6

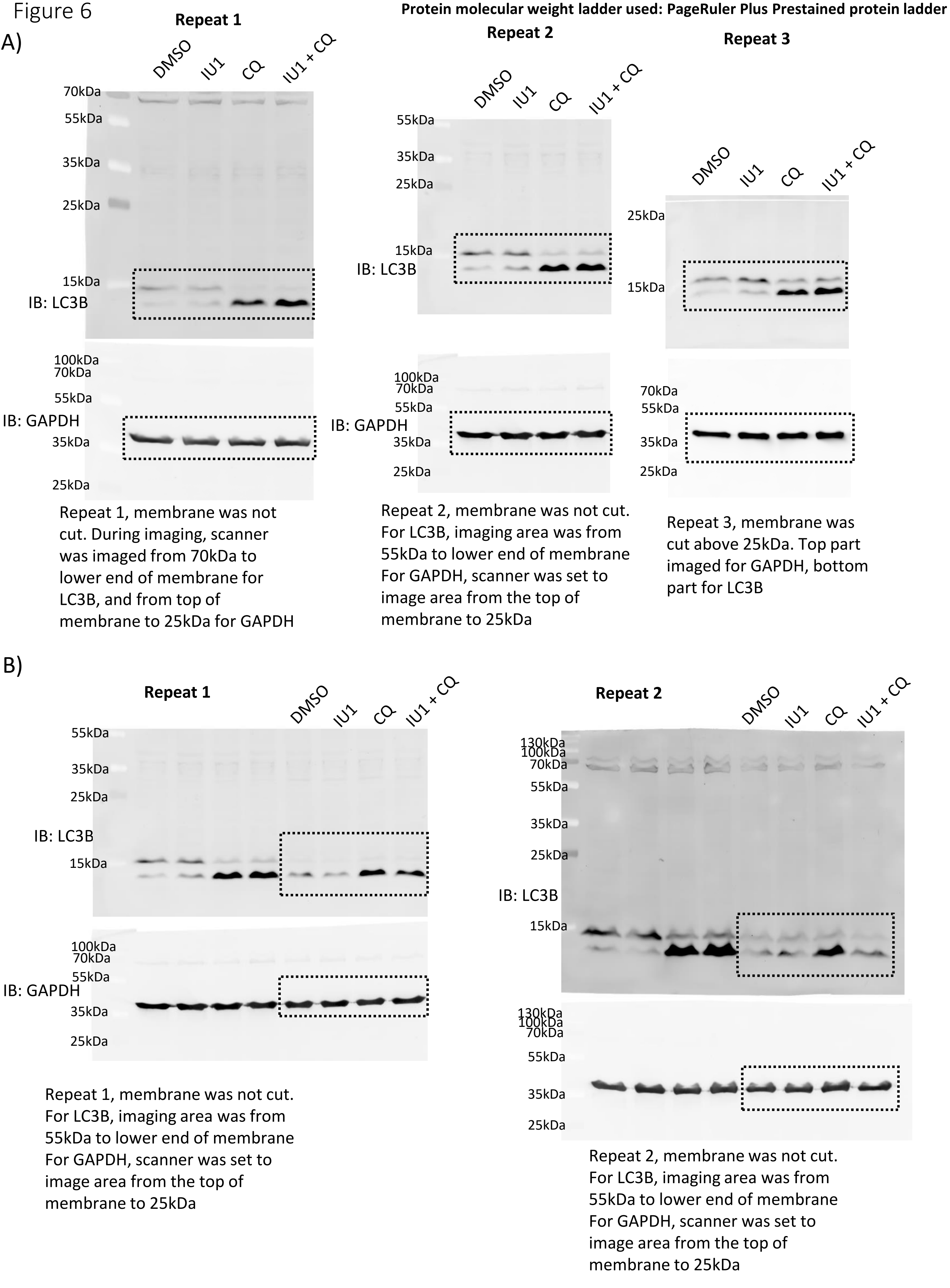
Protein molecular weight ladder used: PageRuler Plus Prestained protein ladder
A)
Repeat 1
DMSO
IU1
CQ
IU1 + CQ
70kDa
55kDa
35kDa
25kDa
15kDa
IB: LC3B
100kDa
70kDa
55kDa
IB: GAPDH
35kDa
25kDa
Repeat 1, membrane was not cut. During imaging, scanner was imaged from 70kDa to lower end of membrane for LC3B, and from top of membrane to 25kDa for GAPDH
Repeat 2
DMSO
IU1
CQ
IU1 + CQ
55kDa
35kDa
25kDa
15kDa
IB: LC3B
100kDa
70kDa
55kDa
IB: GAPDH
35kDa
25kDa
Repeat 2, membrane was not cut. For LC3B, imaging area was from 55kDa to lower end of membrane For GAPDH, scanner was set to image area from the top of membrane to 25kDa
Repeat 3
DMSO
IU1
CQ
IU1 + CQ
25kDa
15kDa
70kDa
55kDa
35kDa
25kDa
Repeat 3, membrane was cut above 25kDa. Top part imaged for GAPDH, bottom part for LC3B
B)
Repeat 1
DMSO
IU1
CQ
IU1 + CQ
55kDa
35kDa
25kDa
IB: LC3B
15kDa
100kDa
70kDa
55kDa
IB: GAPDH
35kDa
25kDa
Repeat 1, membrane was not cut. For LC3B, imaging area was from 55kDa to lower end of membrane For GAPDH, scanner was set to image area from the top of membrane to 25kDa
Repeat 2
DMSO
IU1
CQ
IU1 + CQ
130kDa
100kDa
70kDa
55kDa
35kDa
25kDa
IB: LC3B
15kDa
130kDa
100kDa
70kDa
55kDa
35kDa
25kDa
Repeat 2, membrane was not cut. For LC3B, imaging area was from 55kDa to lower end of membrane For GAPDH, scanner was set to image area from the top of membrane to 25kDa

Figure 6

Protein molecular weight ladder used: PageRuler Plus Prestained protein ladder

B)

Repeat 3

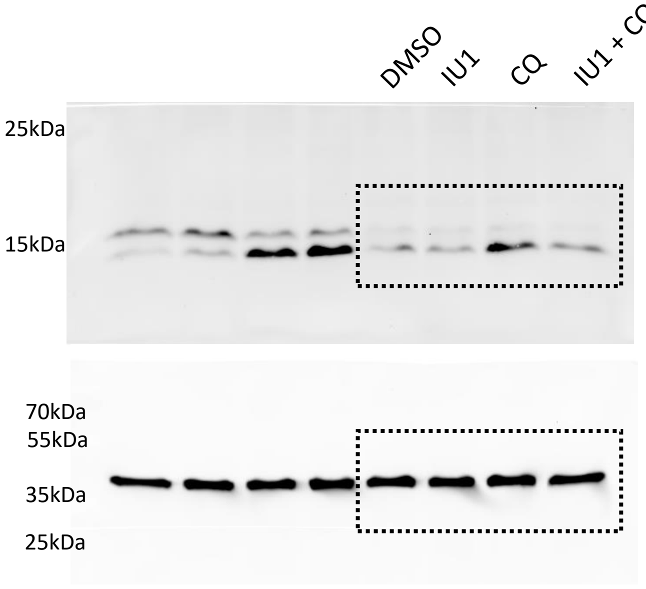

Repeat 3, membrane was cut above 25kDa. Top part imaged for GAPDH, bottom part for LC3B

C)

Repeat 1

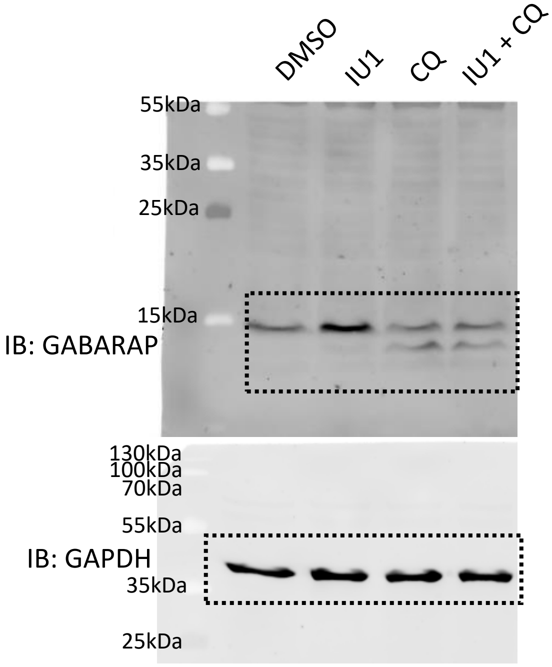

Repeat 1, membrane was not cut. During imaging, scanner was imaged from 55kDa to lower end of membrane for GABARAP, and from top of membrane to 25kDa for GAPDH

Repeat 2

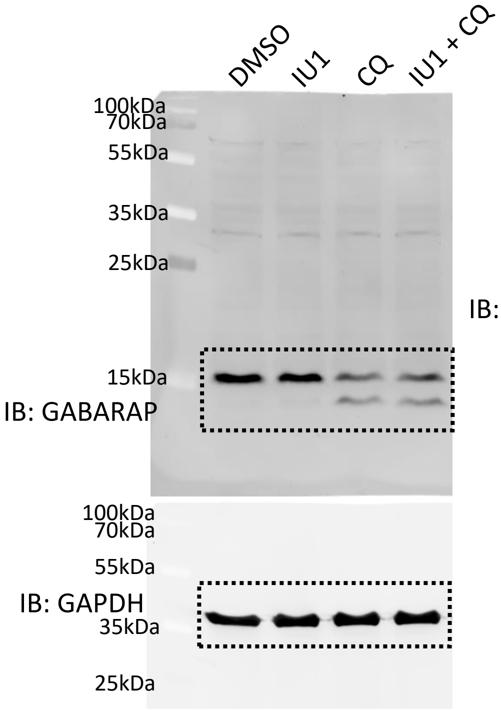

Repeat 2, membrane was not cut. During imaging, scanner was imaged from top of membrane to 25kDa for GAPDH

Repeat 3

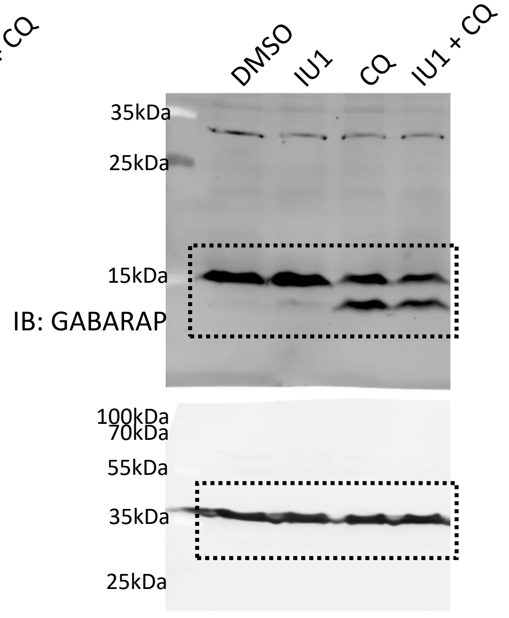

Repeat 3, membrane was not cut. During imaging, scanner was imaged from 35kDa to lower end of membrane for GABARAP, and from top of membrane to 25kDa for GAPDH

Figure 6

Protein molecular weight ladder used: PageRuler Plus Prestained protein ladder

D)

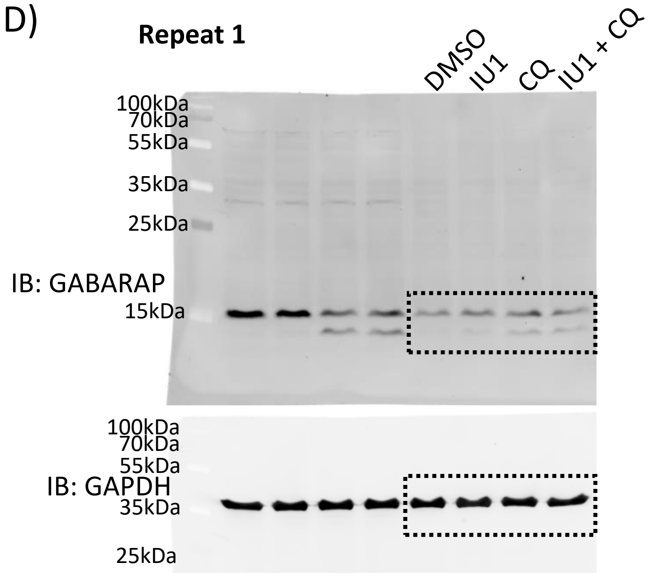

Repeat 1, membrane was not cut. During imaging, scanner was imaged from top of membrane to 25kDa for GAPDH

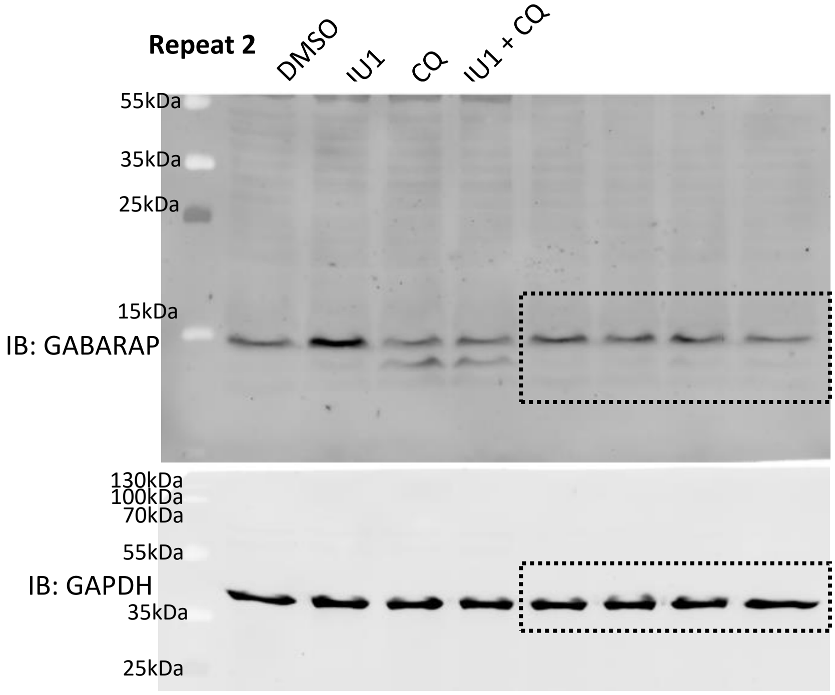

Repeat 2, membrane was not cut. During imaging, scanner was imaged from 55kDa to lower end of membrane for GABARAP, and from top of membrane to 25kDa for GAPDH

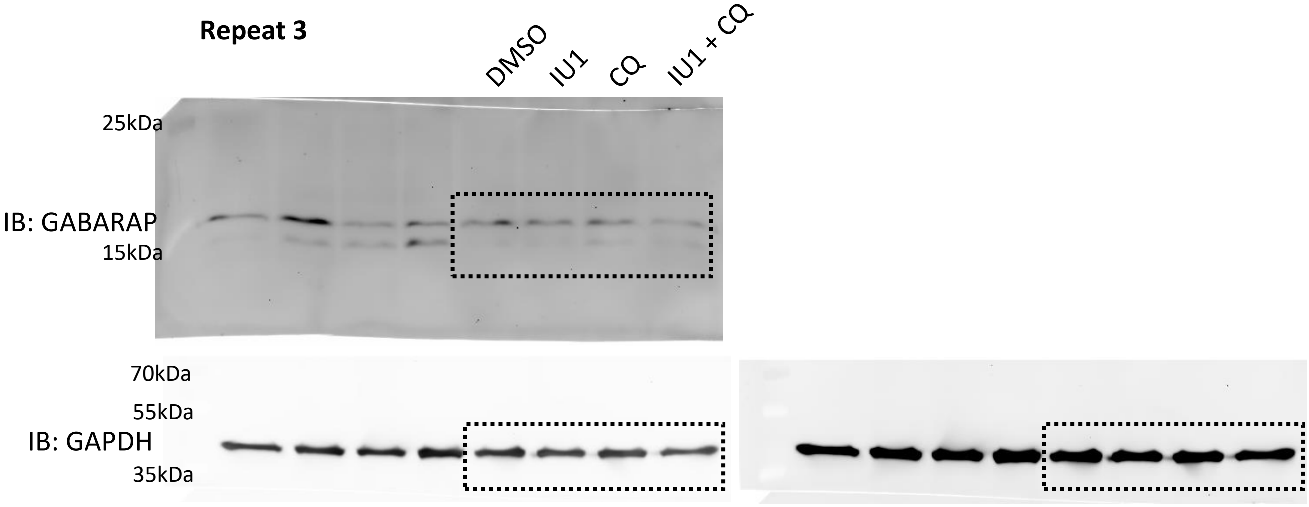

Contrast adjusted to show molecular weight ladder for the image to the left (GAPDH blot)

Repeat 3, membrane was cut above 25kDa MW marker. Lower part was detected with GABARAP antibody, upper part detected with GAPDH. For GAPDH, imaging was done between 70kDa to 35kDa area of membrane

Figure 6

Protein molecular weight ladder used: PageRuler Plus Prestained protein ladder

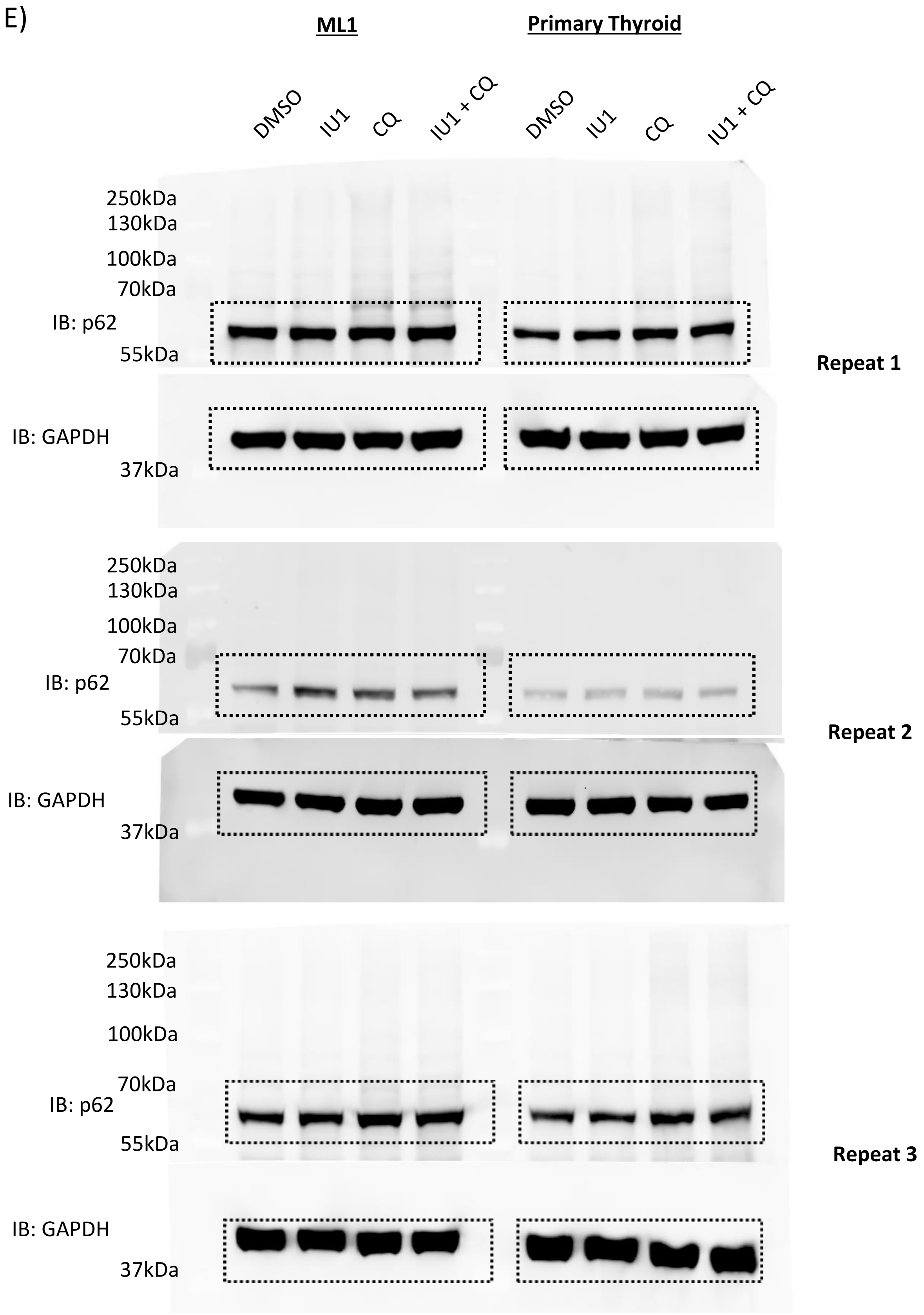

All 3 repeats, membranes were cut below 55kDa, upper half detected with p62 and lower half with GAPDH. First lane from the left and 5th lane from the left are molecular weight ladders to be able to accurately ensure that p62 MW does not change between ML1 and primary thyroid to be able to compare and quantify. Sample loading order same for all 3 repeats, as labelled on the top above repeat 1.

Supplementary Figure 2 **Protein molecular weight ladder used: PageRuler Plus Prestained protein ladder**

A)

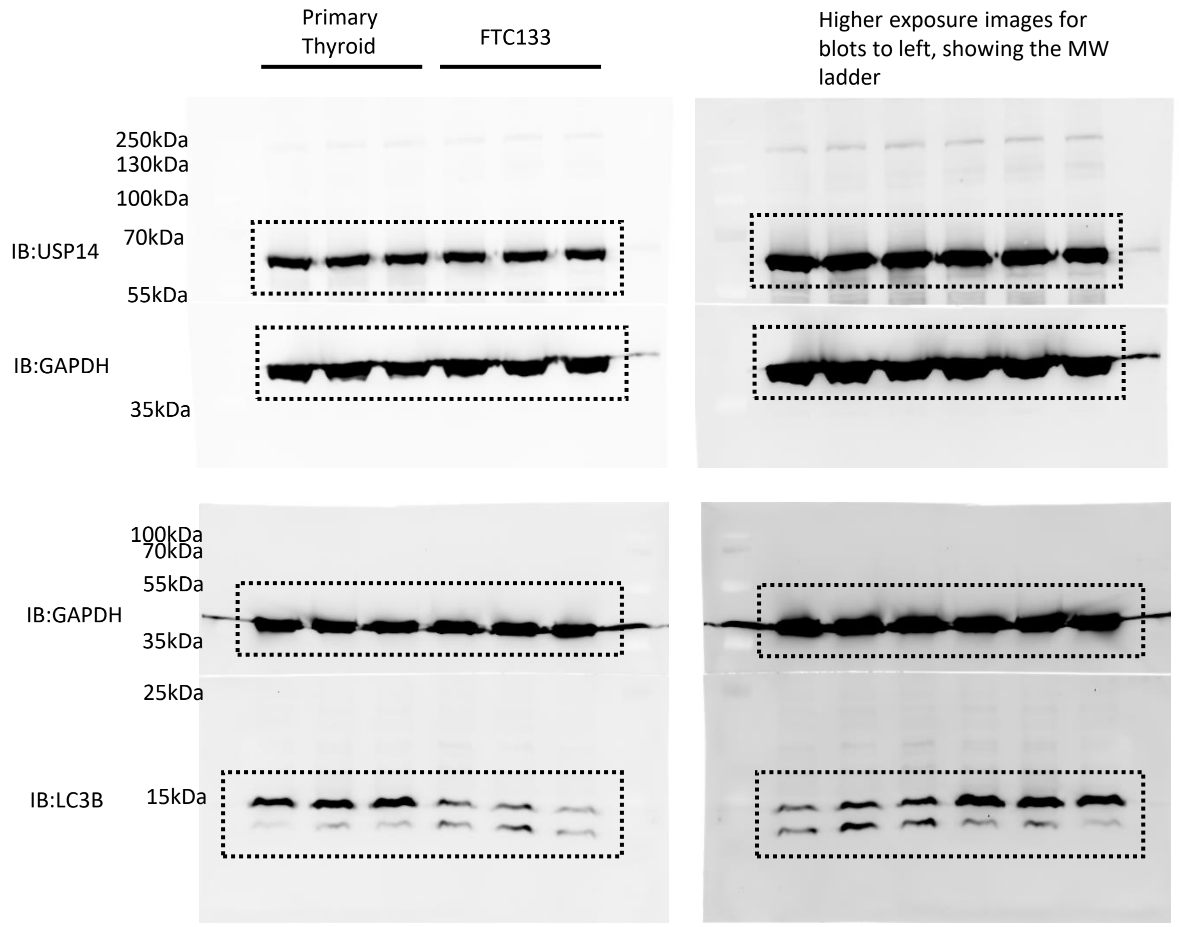

Membranes were cut to detect USP14/ GAPDH. Similary for LC3B/GAPDH

Supplementary Figure 3 **Protein molecular weight ladder used: PageRuler Plus Prestained protein ladder**

A)

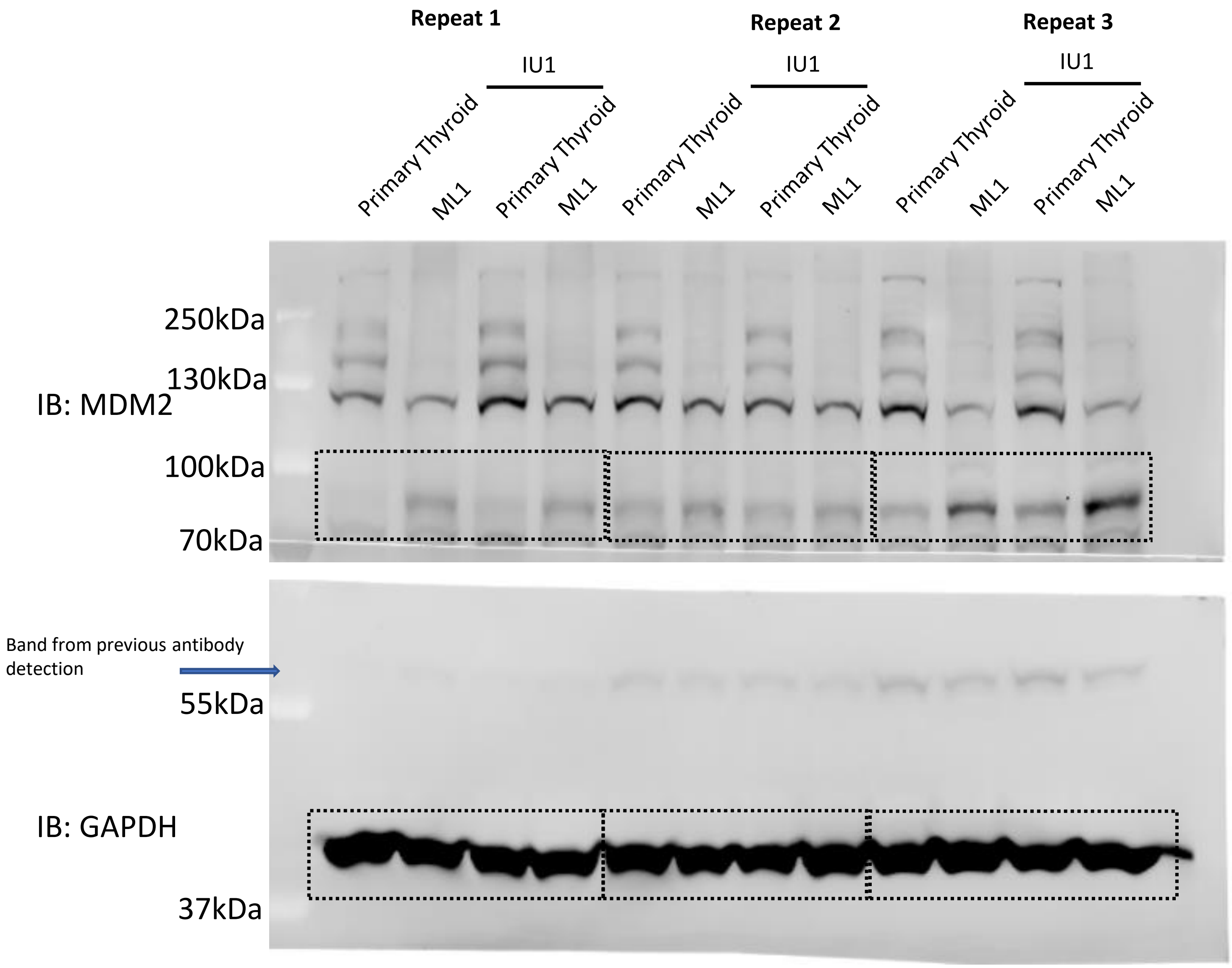

Membrane cut at 70kDa MW ladder, upper membrane shown here detected with MDM2 and lower half of membrane detected with GAPDH antibody.
